# From chromatin to proteostasis: multilevel gene regulation in Mendelian diseases

**DOI:** 10.3389/freae.2026.1944086

**Published:** 2026-09-25

**Authors:** Himanshu Goel, Gunjan Garg

**Affiliations:** 1 Hunter Genetics, Hunter New England Local Health District, Waratah, NSW, Australia; 2 School of Medicine and Public Health, University of Newcastle, Newcastle, NSW, Australia

**Keywords:** DNA methylation, episignatures, gene expression regulation, Mendelian disease, noncoding RNA, proteostasis, RNA splicing, translational regulation

## Abstract

Pathogenic variation can alter gene output at multiple levels, from chromatin accessibility and enhancer-promoter communication to RNA processing, translation and protein turnover. The diagnostic challenge is not to catalogue these layers, but to determine which regulatory mechanism is plausible for a given variant, phenotype, tissue and developmental context. This targeted narrative review synthesises representative Mendelian mechanisms across five linked levels: chromatin, transcription, RNA processing and stability, translation, and post-translational proteostasis. Three cross-cutting principles recur. First, distinct molecular lesions may converge on the same functional bottleneck: altered dosage, timing, localisation or disrupted protein homeostasis. Second, regulatory effects are often cell-type-, isoform-, developmental-stage- and state-specific, so assay and tissue selection are integral to interpretation. Third, a negative result in an accessible surrogate such as blood does not exclude a disease-relevant effect in brain, muscle, liver or stimulated immune cells. From these principles, the review derives a mechanism-directed diagnostic framework that links variant class to orthogonal assays, including DNA methylation profiling, chromatin and reporter assays, RNA sequencing, ribosome-level methods and protein-based studies. The framework is offered as an integrative clinical synthesis rather than a new model of gene regulation, to support variant interpretation, functional-test selection and translational prioritisation in unsolved Mendelian disease.

## 1 Introduction

Gene expression converts genomic information into functional RNA and protein products, but this process is not linear. In eukaryotic cells, gene output is controlled across multiple regulatory layers, including chromatin accessibility (Cain et al., 2011), transcription (Cai and Luse, 1987; Bateman and Paule, 1988; Tan et al., 1995), RNA processing and stability (Aranega and Franco, 2024; Alaei and Kakumani, 2025; Ganguly and Gregory, 2026), translation (Baliga et al., 2025; Jia et al., 2026; Kuse and Ishii, 2025; Petrychenko et al., 2025; Poff et al., 2025), and post-translational regulation (Takla et al., 2023; Zhou et al., 2021; Ball et al., 2004; Nalivaeva and Turner, 2001; Schulze et al., 1987). These layers determine when, where, and how much functional product is generated from a gene (Lelli et al., 2012; Soci et al., 2017). They are tightly interconnected and responsive to developmental cues, environmental stimuli, metabolic states and cellular stress (Dolinoy, 2007). During embryogenesis, selective gene activation drives cellular differentiation despite identical genomic content (Strober et al., 2019), demonstrating that the same genome yields distinct outputs through regulation alone.

This regulatory architecture is clinically important because pathogenic variation may act at any level of gene expression. Coding variants can alter protein structure, but noncoding, synonymous, splice-region, untranslated-region, epigenetic and structural variants may instead alter transcript abundance, RNA processing, translation efficiency or protein turnover. Consequently, phenotype is often determined not only by the affected gene but by the specific regulatory mechanism disrupted (Di Donato et al., 2026; Alyatimi et al., 2026). This complexity affords developmental precision but introduces multiple points of vulnerability, such that disruption at any level can result in Mendelian disease (Nair et al., 2019).

Gene regulation is therefore best interpreted as a context-dependent system rather than a linear DNA-to-protein pathway (Di Donato et al., 2026; Delgado and Gomez-Vela, 2019; Kartha et al., 2022; Menon et al., 2025). Earlier syntheses have established the multilayered architecture of gene regulation. The present review does not claim a new biological model; instead, it translates these established principles into a clinical question: how should the suspected regulatory layer, relevant tissue and expected molecular consequence determine variant interpretation and functional testing? The scope is intentionally selective: representative mechanisms illustrate diagnostic principles rather than provide an encyclopaedic catalogue of all regulatory processes or disorders (Table 1).

**TABLE 1:** Regulatory layers of gene expression and representative disease mechanisms.

Regulatory layer	Core process	Variant/mechanism class	Molecular consequence	Lesion locus	Route to phenotype	Clinical examples
Pre-transcriptional	Chromatin accessibility, DNA methylation, histone modification	Chromatin regulator variants, episignature disorders	Altered gene accessibility and transcriptional potential	Trans	Indirect	*KMT2D*/Kabuki, *EED*-related overgrowth (Goel et al., 2024), *ARID2*-related disorder (Houdayer et al., 2025)
Transcriptional	Promoter recognition, transcription-factor binding, enhancer–promoter interaction	Transcription-factor variants, cohesin defects, enhancer disruption	Altered transcriptional initiation or regulatory specificity	Cis and trans	Direct for the factor; indirect for target genes	*TAF1, ZNF462* (Hau et al., 2025)*, RAD21* (Goel and Parasivam, 2020), *ZRS/SHH*
Post-transcriptional	Splicing, RNA editing, RNA stability, localisation	Splice variants, RNA-binding protein defects, miRNA dosage, noncoding 5′UTR repeat expansion reducing exon junction complex dosage	Altered transcript structure, abundance, or localisation	Cis for splice and UTR lesions; trans for RNA- binding factors	Direct	*SCN1A, HNRNPU* (Hodgson et al., 2025)*, CNOT1* (Vissers et al., 2020), *MIR17HG* (Sharaidin et al.,2013), *RNU4-2* (Nava et al., 2025), *EIF4A3* (Favaro et al., 2014)
Translational	Initiation, elongation, termination, ribosome rescue	Upstream open reading frame variants, codon-usage effects, elongation-factor defects	Altered protein synthesis efficiency or fidelity	Cis for uORF lesions; trans for factors	Direct	*THPO, NIPBL 5′UTR, EEF1A2, EEF2, DDX3X* (Beal et al., 2019)
Post-translational	Folding, modification, trafficking, degradation	Post-translational modification defects, ubiquitin–proteasome/autophagy defects	Altered protein activity, localisation, or turnover	Trans	Direct, or indirect through regulatory substrates	*CSNK2A1*, *TRIP12* (Donoghue et al., 2020), *ALG1-CDG* (Ng et al., 2016), *UBE3A*, *EPG5*

Lesion locus indicates whether the primary lesion lies in a cis-acting element in physical continuity with the affected gene or in a diffusible trans-acting factor. Route to phenotype indicates whether the phenotype follows from altered function of the mutated gene product itself (direct) or from altered expression or handling of downstream targets (indirect).

## 2 Methods

A targeted narrative search was undertaken for publications up to 31 May 2026. PubMed/MEDLINE and Google Scholar were searched using combinations of the terms gene regulation, Mendelian disease, chromatinopathy, enhancer, topologically associating domain, CTCF, cohesin, RNA splicing, *RNU4-2*, nonsense-mediated decay, noncoding RNA, upstream open reading frame, ribosome profiling, specialised ribosome, ubiquitin-proteasome, autophagy, functional genomics, RNA sequencing and proteomics. Reference lists of key reviews and primary disease reports were also examined. Priority was given to human genetic studies with functional evidence, clinically validated diagnostic assays, authoritative mechanistic reviews and representative examples that clarified variant interpretation. Cancer-only studies, purely descriptive expression studies without a defined regulatory mechanism, and redundant examples were generally excluded. Because this is a targeted narrative review rather than a systematic review, no meta-analysis or formal risk-of-bias assessment was performed. For representative mechanisms spanning each regulatory layer, the level of supporting evidence was recorded and is reported in Supplementary Table S1 using four grades: experimentally demonstrated in patient-derived material or an appropriate model system (D); mechanistically inferred or proposed (I); a causal chain containing both demonstrated and inferred links (D/I); and no primary effect expected at that level, or not applicable. The grading refers to the molecular relationships summarised and not to the validity of the gene-disease association, since a mechanism may be well characterised at pathway level while remaining inferred for a given variant.

## 3 Pre-transcriptional regulation (chromatin and three-dimensional genome regulation)

*KMT2D* haploinsufficiency in Kabuki syndrome illustrates a chromatin-level mechanism. *KMT2D*, together with its paralogue *KMT2C*, functions predominantly at enhancers, where these COMPASS-like complexes contribute to H3K4 monomethylation (H3K4me1) and establishment of an enhancer-permissive chromatin state. Enhancer activation is further associated with p300/CBP-dependent H3K27 acetylation. In contrast, promoter-associated H3K4me3 is deposited predominantly by SETD1A/B-containing COMPASS complexes, with KMT2A/B contributing at selected loci. Reduced KMT2D dosage therefore preferentially perturbs enhancer priming and activation at developmentally regulated loci rather than causing a global loss of promoter H3K4me3 (Froimchuk et al., 2017; Lee et al., 2013; Ng et al., 2010). Gene expression is thus regulated upstream of transcription at the chromatin state, and disruption of this layer can then produce widespread transcriptional dysregulation without alteration of coding sequence, contributing to a broad developmental phenotype (Figure 1).

**FIGURE 1:**
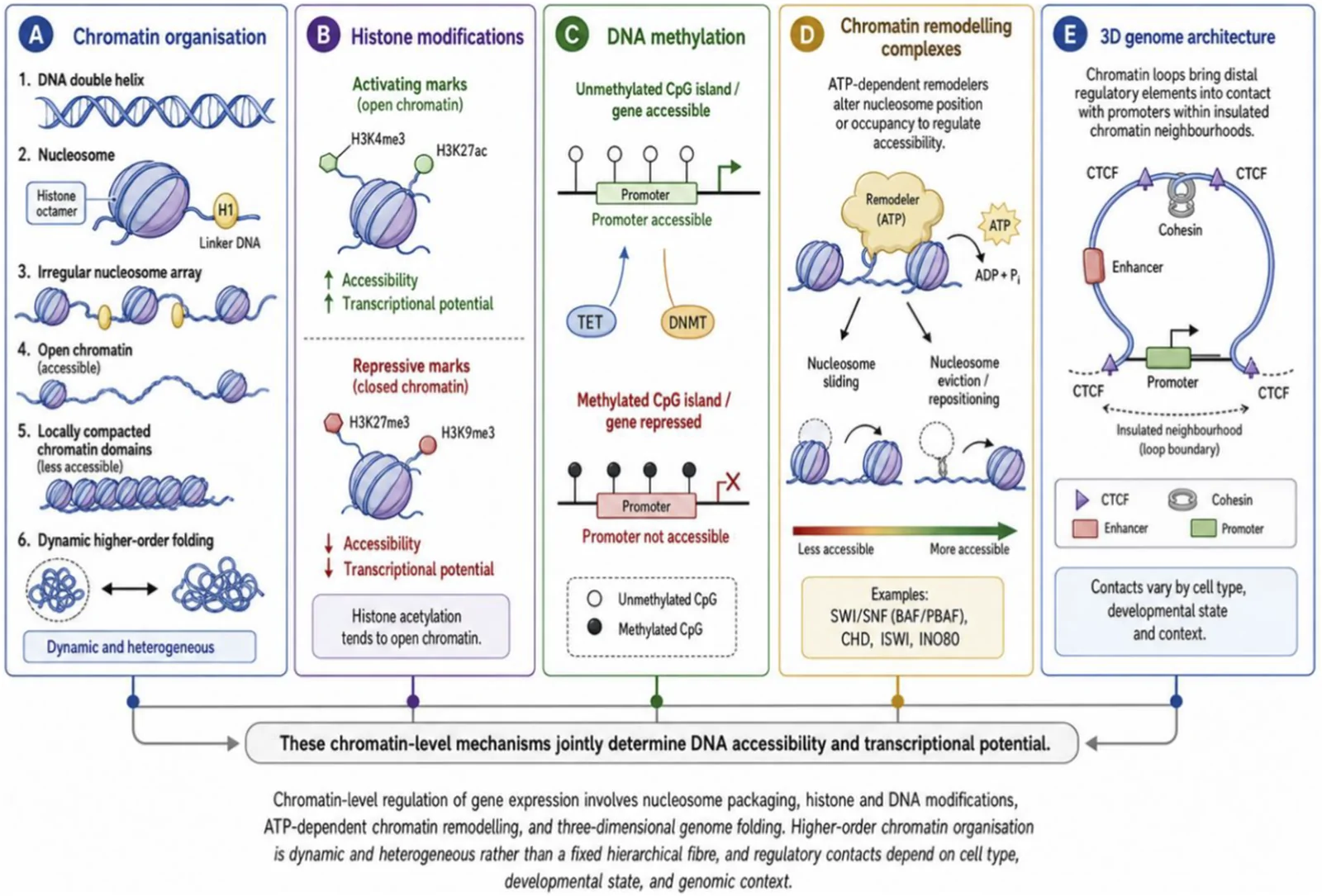
Chromatin-level mechanisms regulating gene accessibility and transcriptional potential. Chromatin-level regulation of gene expression is determined by nucleosome packaging, histone modification, DNA methylation, ATP-dependent chromatin remodelling, and three-dimensional genome organisation. Panel A establishes the substrate as a nested series, in which DNA and histones together constitute the nucleosome, nucleosome arrays constitute chromatin, and chromatin adopts accessible or compacted states; panels B to E depict processes acting on that substrate rather than independent categories, and arrows indicate the direction in which each process modifies accessibility. **(A)** Chromatin organisation ranges from naked DNA and nucleosomes to open chromatin and locally compacted domains; higher-order folding is dynamic and heterogeneous rather than a fixed hierarchical fibre. **(B)** Histone modifications influence accessibility, with activating marks such as H3K4me3 and H3K27ac associated with open chromatin, and repressive marks such as H3K27me3 and H3K9me3 associated with compaction. **(C)** Establishment, maintenance and erasure of DNA methylation by DNMT3A and DNMT3B, DNMT1 and TET-mediated oxidation; methylation at CpG-rich promoters reduces accessibility and transcriptional potential, whereas unmethylated CpG islands are associated with active promoters. **(D)** ATP-dependent chromatin remodelling complexes regulate accessibility by sliding, repositioning, or evicting nucleosomes. **(E)** Three-dimensional genome architecture constrains regulatory interactions by bringing enhancers and promoters into contact within insulated neighbourhoods, with contacts varying by cell type, developmental state, and genomic context.

### 3.1 Chromatin organisation

Genomic DNA is packaged into chromatin, a nucleoprotein structure that regulates DNA accessibility. The basic unit is the nucleosome, in which ∼147 base pairs of DNA wrap around a histone octamer composed of two copies each of H2A, H2B, H3 and H4. Linker DNA connects adjacent nucleosomes, while histone H1 binds linker DNA and supports higher-order compaction; H1 is not part of the core octamer (Studitsky et al., 2016; Venkatesh and Workman, 2015).

Euchromatin is relatively open, enriched in activating histone modifications, and accessible to transcription factors and RNA polymerase II. In contrast, heterochromatin is densely compacted and associated with repressive epigenetic marks that restrict transcription. Heterochromatin may be constitutive, as seen at centromeres and telomeres, or facultative, as exemplified by the inactive X chromosome. Dynamic transitions between these states enable context-dependent gene regulation.

### 3.2 Histone modifications

Histone tails undergo reversible post-translational modifications, including acetylation, methylation, phosphorylation, ubiquitination and SUMOylation. These marks regulate transcription by changing nucleosome stability, altering DNA–histone interactions, or creating binding platforms for effector proteins.

Histone acetylation, deposited by histone acetyltransferases, neutralises lysine charge and promotes open chromatin. Histone deacetylases remove acetyl groups and favour chromatin compaction and transcriptional repression (Sheikh, 2014). Histone methylation has residue-specific and state-specific effects, and the writers of each state differ. H3K4me3 marks active promoters, and is deposited principally by SETD1A and SETD1B and by KMT2A and KMT2B, whereas H3K4me1 marks primed and active enhancers and is deposited principally by KMT2C and KMT2D. H3K27me3 is associated with Polycomb-mediated repression (PRC), and H3K9me3 marks constitutive heterochromatin (Yu et al., 2025; Zhou et al., 2019; Igolkina et al., 2019; Nicetto et al., 2019; Nicetto and Zaret, 2019). These marks are written, erased and interpreted by specialised chromatin proteins, allowing transcriptional states to be stable yet reversible. Collectively, combinations of histone modifications are often referred to as the histone code, in which coordinated modification patterns integrate multiple regulatory inputs to determine chromatin state and transcriptional output. Disruption of PRC2 components, such as EED*,* alters Polycomb-mediated repression and causes overgrowth syndromes (Goel et al., 2024).

### 3.3 DNA methylation

DNA methylation occurs mainly at CpG dinucleotides and is a key mechanism of transcriptional repression. CpG-rich promoters are usually unmethylated in active genes, allowing transcription-factor binding. In contrast, promoter methylation can inhibit transcription-factor access and recruit methyl-CpG-binding proteins, including MeCP2 and related factors. These proteins assemble corepressor complexes containing histone deacetylases and other chromatin modifiers, reinforcing chromatin compaction and transcriptional silencing.

DNA methylation patterns are established by DNMT3A and DNMT3B, maintained after DNA replication by DNMT1, and actively modified through TET-mediated oxidation of 5-methylcytosine. Functionally, DNA methylation contributes to genomic imprinting, X-chromosome inactivation, transposable-element repression and lineage-specific gene regulation. Global hypomethylation can promote genomic instability, while focal promoter hypermethylation can silence developmentally important genes. In neurodevelopmental disorders, disruption of methylation machinery can impair neuronal maturation and activity-dependent gene regulation (Zhang et al., 2023; Clouaire and Stancheva, 2008; Ehrlich, 2019).

Methylation state can also be allele-specific, and where it is, the same sequence can be compatible with health or with disease depending on which allele carries the mark. At 11p15.5, gain of methylation at the *H19*/*IGF2* imprinting control region and loss of methylation at *KCNQ1OT1* produce Beckwith-Wiedemann and Silver-Russell phenotypes that are opposite in direction, while the underlying sequence may be unchanged. At 15q11-q13, the parental origin of a deletion or of uniparental disomy determines whether Prader-Willi or Angelman syndrome results, and *UBE3A* is silenced on the paternal allele in neurons but not in other tissues, so the phenotype depends jointly on allele, tissue and developmental stage. Primary epimutation and cis-acting sequence variants that cause secondary epimutation can be distinguished by testing whether the methylation defect segregates with a haplotype. Methylation is therefore best treated as an allele-level rather than a locus-level property, and the diagnostic question is which allele carries the mark, in which tissue, and at what developmental stage (Monk et al., 2019).

### 3.4 Chromatin remodelling complexes

ATP-dependent chromatin remodelling complexes reposition, evict or restructure nucleosomes to regulate DNA accessibility. By altering nucleosome position or composition, they control whether promoters, enhancers and other regulatory elements are available to the transcriptional machinery. These complexes utilise the energy derived from ATP hydrolysis to disrupt histone–DNA contacts, enabling nucleosome sliding, eviction, repositioning, or histone variant exchange. These complexes are broadly classified into four major families: SWI/SNF, ISWI, CHD, and INO80, each defined by characteristic domain architecture and biological roles (Table 2). While all four families utilise ATP-driven DNA translocation, they are functionally specialised: SWI/SNF complexes promote chromatin opening and activation, ISWI complexes organise nucleosome spacing, CHD proteins integrate epigenetic signals into developmental gene regulation, and INO80 complexes coordinate histone variant dynamics and genome stability.

**TABLE 2:** Chromatin remodelling complexes.

Remodelling family	Main function	Clinical relevance
SWI/SNF/BAF/PBAF	Opens chromatin by sliding or destabilising nucleosomes; important for lineage specification and cell identity	Pathogenic variants cause Coffin–Siris syndrome, Nicolaides–Baraitser syndrome and other neurodevelopmental disorders; somatic variants occur in several cancers (Houdayer et al., 2025; Santen et al., 2012). *ARID2* provides a useful PBAF-related example, particularly where episignature data are discussed (Houdayer et al., 2025)
ISWI	Regulates nucleosome spacing and higher-order chromatin organisation	Pathogenic variants in *SMARCA5* and *RSF1* have been reported in neurodevelopmental disorders (Li et al., 2021)
CHD	Uses chromodomains to integrate histone-methylation signals with nucleosome remodelling	*CHD7* causes CHARGE syndrome, *CHD8* is associated with autism and macrocephaly (Sugathan et al., 2014), and *CHD2* causes developmental and epileptic encephalopathy (Lamar and Carvill, 2018)
INO80	Mediates histone-variant exchange, transcriptional regulation, replication stress responses and DNA repair	*YY1* interacts with INO80-related remodelling machinery; pathogenic YY1 variants cause a syndromic neurodevelopmental disorder with intellectual disability, growth restriction and congenital anomalies (Ayala et al., 2018)

### 3.5 Regulatory elements and 3D genome architecture

Gene regulation is shaped by three-dimensional genome organisation as well as linear sequence. Cohesin is loaded onto chromatin and extrudes DNA into progressively larger loops until it encounters convergently oriented CTCF sites or other barriers. This loop-extrusion process enriches enhancer-promoter contacts within insulated neighbourhoods and contributes to topologically associating domains (TADs), although TADs are population-averaged structures rather than rigid compartments. Pathogenic structural variants can delete a boundary, invert CTCF orientation, reposition an enhancer or change genomic distance, thereby causing enhancer adoption, enhancer disconnection or ectopic repression. The interpretive unit is therefore not merely whether a variant overlaps a TAD, but whether it plausibly changes a disease-relevant regulatory contact in the appropriate cell type and developmental window (Liu et al., 2020; Yao et al., 2015; Spielmann and Mundlos, 2016).

TAD boundaries are enriched for architectural proteins such as CTCF and cohesin, which stabilise chromatin looping. Disruption of this architecture can cause disease through ectopic enhancer activity, gene silencing or altered developmental gene dosage.

Enhancer activity is highly cell-type and developmental-stage specific and integrates environmental and signalling inputs. Pathogenic disruption of enhancers or their spatial interactions is a well-established cause of Mendelian disease that leaves the coding sequence intact. In limb development, point mutations within the ZRS enhancer of *SHH* lead to ectopic expression in the anterior limb bud and result in preaxial polydactyly (VanderMeer et al., 2014). Structural variants in distant regulatory elements controlling *SHH* expression can also cause holoprosencephaly due to impaired midline forebrain patterning (Jeong et al., 2008). Similarly, deletions of the HS-40 enhancer located upstream of the α-globin genes reduce expression of *HBA1* and *HBA2*, resulting in α-thalassaemia (Coelho et al., 2010). Many active enhancers produce short, bidirectional enhancer RNAs (eRNAs), which may contribute to stabilisation of enhancer–promoter interactions (Lewis et al., 2019).

Insulators restrict inappropriate enhancer–promoter communication and preserve regulatory specificity. The zinc-finger protein CTCF plays a central role in insulator function and TAD boundary formation. Loss or disruption of CTCF-binding sites alters chromatin topology, enabling enhancer hijacking and aberrant gene activation. Deletions or rearrangements that disrupt CTCF-defined TAD boundaries can expose neighbouring developmental genes, such as *PAX3* or *WNT6*, to enhancers that are normally restricted to *EPHA4*. This results in ectopic gene activation during limb development and leads to congenital anomalies, including polydactyly and limb malformations (Spielmann and Mundlos, 2016). From a diagnostic perspective, insulator dysfunction underscores the importance of considering three-dimensional genome organisation in genetic disease: such defects are typically caused by structural variants rather than single-nucleotide changes and may not be detected by exome sequencing. Whole-genome sequencing, especially long-read sequencing and analysis of chromatin architecture, is therefore increasingly important for identifying pathogenic variants that disrupt regulatory boundaries.

## 4 Transcriptional regulation

*TAF1*-related neurodevelopmental disorder illustrates disease caused by impaired promoter recognition and pre-initiation complex function rather than alteration of a single downstream structural protein (Crombie et al., 2024; Bernardini et al., 2023; O’Rawe et al., 2015).

Promoter recognition, pre-initiation complex assembly, transcription-factor binding, enhancer-promoter communication, signal-responsive transcription and chromatin-mediated repression coordinate RNA polymerase II activity. Disruption of these processes alters transcriptional output and contributes to neurodevelopmental and multisystem disease (Figure 2).

**FIGURE 2:**
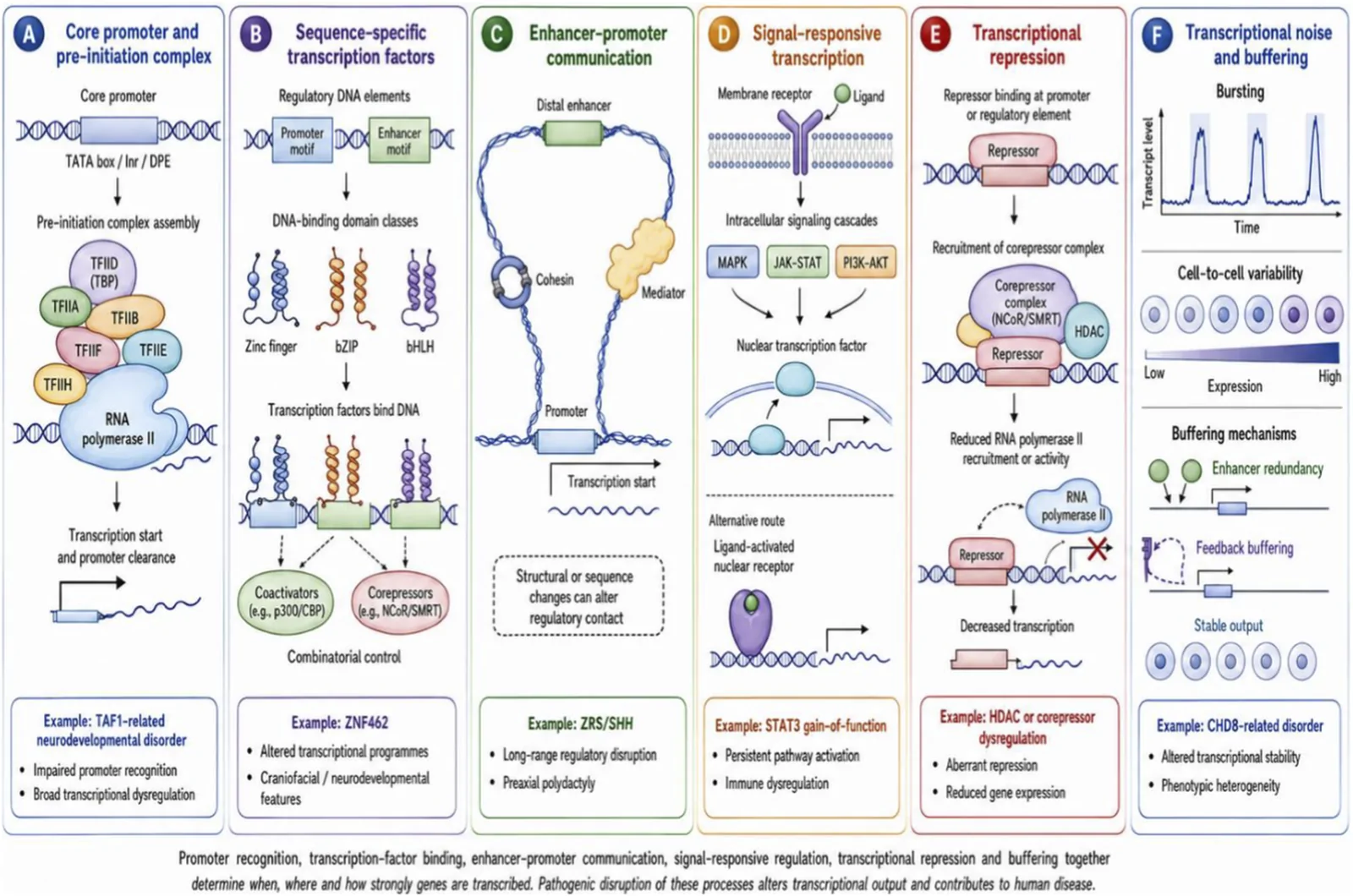
Transcriptional regulation and its perturbation in human disease. Transcriptional output is controlled by promoter recognition, sequence-specific transcription-factor binding, enhancer-promoter communication, signal-responsive transcription, transcriptional repression, and buffering of transcriptional noise. **(A)** Core promoter elements recruit the pre-initiation complex and RNA polymerase II to initiate transcription. Disruption of promoter recognition or pre-initiation complex assembly can alter transcriptional output and contribute to human disease. **(B)** Sequence-specific transcription factors bind promoter and enhancer motifs through distinct DNA-binding domains and regulate transcription through coactivator and corepressor recruitment. Pathogenic disruption of these interactions can alter gene-expression programmes and contribute to disease. **(C)** Enhancers communicate with promoters through chromatin looping mediated by factors such as cohesin and Mediator, and disruption of these long-range contacts can alter developmental gene expression. **(D)** Signal-responsive transcription links extracellular cues to nuclear transcriptional responses through intracellular signalling cascades or ligand-activated nuclear receptors. **(E)** Repressors bound at promoters or regulatory elements recruit corepressor complexes, including NCoR/SMRT and histone deacetylases, thereby reducing RNA polymerase II recruitment or activity and decreasing transcription. **(F)** Transcription is intrinsically burst-like and variable between cells, but buffering mechanisms such as enhancer redundancy and feedback regulation promote stable output. Pathogenic disruption of these processes alters transcriptional programmes and contributes to human disease.

### 4.1 Core promoter and pre-initiation complex

Transcription by RNA polymerase II begins at the core promoter, the minimal DNA region required for transcription initiation. It usually spans approximately −40 to +40 base pairs around the transcription start site and may contain elements such as the TATA box, Initiator sequence, TFIIB recognition element and downstream promoter element. These motifs are recognised by general transcription factors, which assemble with RNA polymerase II to form the pre-initiation complex. This complex includes TFIIA, TFIIB, TFIID, TFIIE, TFIIF and TFIIH. TFIID, composed of the TATA-binding protein and TBP-associated factors, helps recognise promoter elements, while TFIIH unwinds DNA and phosphorylates the RNA polymerase II C-terminal domain, enabling promoter clearance and transition to elongation.

Pathogenic variants in *TAF1* provide a direct example of disease arising from disruption of this machinery. *TAF1* encodes the largest subunit of TFIID and has kinase, acetyltransferase and regulatory functions that link promoter recognition with local chromatin state and transcriptional activation. These activities support recruitment and stabilisation of the transcriptional machinery at promoters. Pathogenic *TAF1* variants cause a syndromic neurodevelopmental disorder characterised by intellectual disability, dysmorphic features and neurological impairment. The phenotype reflects broad transcriptional dysregulation caused by impaired pre-initiation complex function, rather than disruption of a single downstream pathway (Crombie et al., 2024; Bernardini et al., 2023; O’Rawe et al., 2015).

### 4.2 Sequence-specific transcription factors

Sequence-specific transcription factors bind to defined DNA motifs within promoters and enhancers through conserved DNA-binding domains, including zinc fingers, helix–turn–helix motifs, leucine zipper (bZIP) domains, and basic helix–loop–helix (bHLH) domains. By recruiting coactivators, corepressors and chromatin-modifying enzymes, they regulate transcriptional initiation, elongation and repression in a cell-type- and developmental-stage-specific manner.

Gene expression is governed by combinatorial control, in which multiple transcription factors cooperate at regulatory elements to integrate developmental, environmental and signalling inputs. Small changes in transcription factor dosage or binding specificity can therefore produce broad effects on downstream gene-expression networks. Pathogenic variants in *ZNF462*, for example, cause a neurodevelopmental disorder with craniofacial anomalies and corpus callosum abnormalities, reflecting impaired transcriptional programme establishment (Weiss et al., 2017). Although transcription factors act at promoters, many genes are controlled primarily by distal enhancers, which requires precise enhancer-promoter communication.

### 4.3 Enhancer–promoter communication

Enhancers regulate transcription by physically interacting with target promoters through chromatin looping. These interactions are facilitated by architectural proteins and cohesin complexes, allowing distal regulatory elements to influence RNA polymerase II recruitment, promoter activation and transcriptional output. Most developmental genes are controlled by multiple enhancers that act combinatorially to integrate spatial, temporal and signal-dependent inputs. Disruption of enhancer–promoter communication can cause disease without altering coding sequence. This may occur through variants within enhancer elements, altered transcription-factor binding, impaired chromatin looping, or disruption of topological domain architecture (Petrascheck et al., 2005).

The SHH limb enhancer, the zone of polarising activity regulatory sequence (ZRS), provides a classic example. The ZRS lies more than 1 Mb from the SHH promoter but drives spatially restricted posterior limb bud expression. Pathogenic *ZRS* variants can cause ectopic anterior SHH expression, resulting in preaxial polydactyly, while loss of enhancer activity can impair normal developmental patterning (Lettice et al., 2002; Gurnett et al., 2007; Liu et al., 2017). This example illustrates that transcriptional precision depends not only on promoter sequence and transcription factors, but also on correct long-range regulatory communication.

### 4.4 Signal-responsive transcriptional regulation

Signal-responsive transcription links extracellular cues to gene expression through intracellular signalling pathways that converge on transcriptional regulators, enabling rapid and context-dependent modulation of transcriptional programmes. Canonical pathways, including MAPK, JAK-STAT and PI3K-AKT, regulate transcription by post-translational modification of transcription factors and co-regulators (e.g., phosphorylation, acetylation, ubiquitination). These modifications alter DNA-binding affinity, nuclear localisation, protein stability and interactions with coactivator or corepressor complexes, thereby directly modulating transcriptional output without changes to DNA sequence.

Nuclear receptors provide a direct mechanism of signal integration. As ligand-activated transcription factors, they respond to hormones such as glucocorticoids, thyroid hormone and retinoic acid, undergoing conformational changes that promote coactivator recruitment and binding to hormone response elements. Transcriptional output therefore reflects the timing, strength and interaction of multiple signalling inputs within a specific chromatin context.

Gain-of-function variants in *STAT3* cause persistent pathway activation and aberrant transcription of immune and proliferative genes, producing early-onset immune dysregulation with autoimmunity, lymphoproliferation and increased susceptibility to infection (Desoki et al., 2026). This illustrates that transcriptional disease may arise from sustained upstream signalling abnormalities rather than defects in core transcriptional machinery.

### 4.5 Transcriptional repression mechanisms

Transcriptional repression restricts gene expression in a context-dependent manner. Sequence-specific repressors can bind regulatory DNA elements directly, compete with activators or recruit corepressor complexes that reduce promoter and enhancer accessibility. These complexes often include histone deacetylases and repressive chromatin modifiers, which promote chromatin compaction and limit transcription-factor access.

Repression can also act directly on the transcriptional machinery by reducing pre-initiation complex assembly, limiting RNA polymerase II recruitment or stabilising long-range chromatin interactions that silence gene clusters. Together, these mechanisms ensure that genes inappropriate for a given cell type, developmental stage or signalling state remain inactive (Ahmad et al., 2025; Contreras and Perea-Resa, 2024).

Disruption of transcriptional repression leads to inappropriate gene activation. A prototypical example is *MECP2*, which encodes a methyl-CpG-binding protein that represses transcription by recruiting corepressor complexes to methylated DNA. Loss of function results in widespread transcriptional dysregulation in neurons and manifests clinically as Rett syndrome, underscoring the critical role of transcriptional repression in maintaining stable gene expression in the central nervous system (Ahmad et al., 2025; Choi et al., 2025).

### 4.6 Transcriptional noise and stochasticity

Transcription is inherently stochastic. Genes are transcribed in bursts, switching between active and inactive states rather than continuously, and this variability arises from chance interactions among small numbers of molecules. It is influenced by both intrinsic factors, such as promoter architecture and transcription-factor binding, and extrinsic factors, such as signalling state, metabolism and chromatin context (Zhang et al., 2024; Jenkins et al., 2024).

Gene regulation therefore controls not only how much a gene is expressed, but also how consistent that expression is across cells. Cells use multiple buffering mechanisms, including chromatin regulation, feedback loops and enhancer redundancy, to stabilise transcriptional output and maintain reliable gene expression programmes. Stochasticity can be beneficial, enabling developmental flexibility and allowing cells to adopt different fates. However, when variability becomes excessive or poorly controlled, it can disrupt gene expression programmes, impair development and contribute to disease, particularly in neurodevelopmental disorders and cancer (Bax et al., 2026).

A clinically relevant example of dysregulated transcriptional stability is seen in disorders associated with pathogenic variants in *CHD8*. *CHD8* encodes a chromatin remodelling protein that regulates promoter accessibility and stabilises transcriptional output of neurodevelopmental genes. Loss-of-function variants are associated with autism spectrum disorder, macrocephaly, and developmental delay. Disruption of CHD8 may alter both mean gene expression and transcriptional stability across cells, contributing to phenotypic heterogeneity (Di Donato et al., 2026; Sugathan et al., 2014).

## 5 Post-transcriptional regulation (RNA processing, stability and localisation)

*SCN1A* splice-disrupting variants illustrate how abnormal exon definition can generate premature termination codons, activate nonsense-mediated decay and reduce Nav1.1 dosage in Dravet syndrome.

Post-transcriptional regulation encompasses the processes that act on RNA following synthesis but before translation. These include 5′ capping, splicing, 3′ polyadenylation, RNA editing, nuclear export, subcellular localisation, RNA stability control and RNA surveillance. Together, these mechanisms determine whether a transcript is correctly processed, stable, localised and available for protein synthesis. This regulatory layer functions as a series of quality-control and decision checkpoints, governing RNA fate rather than transcriptional initiation. It expands regulatory capacity by enabling a single gene to generate multiple functional outputs and allows rapid modulation of gene expression independent of DNA-level changes.

### 5.1 RNA processing

RNA processing converts nascent pre-mRNA into a mature, translation-competent transcript through a series of coordinated modifications, including 5′ capping, splicing, and 3′ polyadenylation. These steps occur co-transcriptionally and are closely coupled to RNA polymerase II, ensuring efficient and accurate transcript maturation. RNA processing is a key regulatory layer that determines coding sequence integrity, transcript stability, nuclear export, and translational efficiency. Alternative splicing and alternative polyadenylation further expand transcript diversity, allowing a single gene to generate multiple functionally distinct isoforms in a tissue- and developmental stage–specific manner.

Disruption of RNA processing can alter transcript structure through exon skipping, intron retention, frameshifts, premature termination codons or aberrant isoforms. These defects often lead to nonsense-mediated decay or production of dysfunctional proteins, even when transcription itself is preserved.

#### 5.1.1 5′ capping

Shortly after transcription initiation, nascent pre-mRNA undergoes 5′ capping through the addition of a 7-methylguanosine cap via 5′–5′ triphosphate linkage. This chemically distinct structure is recognised by cap-binding proteins. The 5′ cap protects the transcript from exonucleolytic degradation, promotes nuclear export and enables efficient translation initiation. Defective capping or cap recognition can reduce transcript stability, impair nuclear export and diminish translation efficiency. Pathogenic variants directly affecting core capping enzymes have not been clearly established as a common cause of Mendelian disease, suggesting that this process may be highly constrained. However, disruption of cap-dependent translation is clinically relevant. Variants in *EIF4E*, a key cap-binding protein, have been associated with neurodevelopmental disorders, including autism spectrum disorder, through altered cap-dependent translation and dysregulated neuronal protein synthesis (Kachaev et al., 2020; Amorim et al., 2018).

This distinction highlights that while cap formation itself is highly conserved and likely intolerant to disruption, downstream utilisation of the cap structure represents a clinically relevant vulnerability.

#### 5.1.2 Pre-mRNA splicing

Most human genes contain introns that must be precisely removed to generate a functional mRNA. Splicing is mediated by the spliceosome, a dynamic ribonucleoprotein complex composed of small nuclear ribonucleoproteins, including U1, U2, U4, U5 and U6, together with associated proteins. Spliceosome assembly occurs in a stepwise manner and is tightly coordinated with transcription by RNA polymerase II.

Accurate splicing requires recognition of conserved sequence elements, including the 5′ splice site, 3′ splice site, and branch point sequence. These are interpreted together with auxiliary regulatory motifs, such as exonic and intronic splicing enhancers or silencers, which are bound by RNA-binding proteins, including heterogeneous nuclear ribonucleoproteins (Senn and Hoskins, 2024; Lee and Stevens, 2016). Disruption of splicing is a major mechanism of human disease. Variants affecting canonical splice sites, branch points or regulatory elements can cause exon skipping, intron retention or activation of cryptic splice sites, often leading to frameshifts, premature termination codons and nonsense-mediated decay. Importantly, variants predicted at the DNA level to be missense or synonymous may exert their pathogenic effect primarily through altered splicing. RNA-based diagnostics, particularly RNA sequencing, are increasingly revealing pathogenic splicing defects in previously unresolved genetic disorders. This highlights pre-mRNA splicing as a critical regulatory checkpoint linking genomic variation to functional gene output (Liu et al., 2022).

#### 5.1.3 3′ polyadenylation

Formation of the 3′ end of mRNA is a tightly regulated co-transcriptional process. After transcription of a polyadenylation signal sequence, most commonly AAUAAA, a multiprotein complex cleaves the pre-mRNA downstream of this site. Poly(A) polymerase then adds a polyadenosine tail, which is bound by poly(A)-binding proteins to stabilise the transcript and coordinate downstream processing. The poly(A) tail protects mRNA from exonucleolytic degradation, facilitates nuclear export and promotes efficient translation through interaction with cap-binding complexes and ribosome recycling. Gradual shortening of the poly(A) tail (deadenylation) is a key step in regulated mRNA decay. Alternative polyadenylation allows a single gene to generate transcripts with different 3′ untranslated regions, altering regulatory element content such as microRNA and RNA-binding protein binding sites.

Pathogenic variants affecting the polyadenylation signal can impair 3′ end formation, resulting in aberrant transcript processing and reduced gene expression. A patient with β-thalassaemia was found to carry a single nucleotide substitution (T→C) within the canonical polyadenylation signal (AATAAA) of the human β-globin gene, resulting in AACAAA. This mutation impaired normal 3′-end cleavage and polyadenylation. As a result, transcription read through the usual poly(A) site and produced an abnormally elongated ∼1500-nt β-globin mRNA, with polyadenylation occurring ∼900 bp downstream at a cryptic AATAAA sequence in the 3′ flanking region. The inefficient processing reduced normal β-globin mRNA levels and caused a β-thalassaemia phenotype (Chen et al., 2021; Orkin et al., 1985). Through regulated cleavage and polyadenylation, cells fine-tune mRNA stability, localisation, and translational capacity, adding another critical layer of post-transcriptional gene control (Danckwardt et al., 2008).

#### 5.1.4 RNA surveillance and nonsense-mediated decay

Following RNA processing, cells employ surveillance mechanisms to ensure that only correctly processed transcripts are translated. Nonsense-mediated decay (NMD) is a major quality-control pathway that degrades mRNAs containing premature termination codons, which may arise from nonsense variants, frameshifts or aberrant splicing.

During the pioneer round of translation, transcripts are assessed for features indicating premature termination, including exon-exon junction complexes downstream of the stop codon. Recognition of an abnormal termination event activates conserved NMD factors, including UPF1, UPF2 and UPF3B, leading to transcript degradation. NMD also regulates a subset of normal transcripts, contributing to gene-expression control during development and stress responses.

The clinical effect of NMD depends on context. Degradation of mutant transcripts may prevent production of truncated toxic proteins, but can also cause loss of partially functional transcripts and result in haploinsufficiency. Conversely, escape from NMD may permit dominant-negative or toxic gain-of-function effects. Loss-of-function variants in *UPF3B*, a core component of the NMD pathway, are associated with X-linked neurodevelopmental disorders characterised by intellectual disability, autism spectrum features and variable neurological involvement, highlighting the importance of RNA surveillance in neuronal gene-expression control (Yi et al., 2022; Jolly et al., 2013). NMD also modifies disease severity in many genetic disorders. In dystrophinopathies, variants that abolish dystrophin production typically cause Duchenne muscular dystrophy, whereas variants preserving partially functional protein are associated with the milder Becker phenotype. Thus, RNA surveillance links variant consequence, transcript stability and clinical expression (Koeks et al., 2017; Beroud et al., 2004).

The exon junction complex can itself be the locus of Mendelian disease. *EIF4A3* encodes eIF4AIII, the DEAD-box RNA helicase that nucleates the exon junction complex approximately 20–24 nucleotides upstream of exon-exon junctions and provides the platform through which UPF3B and UPF2 link splicing history to nonsense-mediated decay. Richieri-Costa-Pereira syndrome, an autosomal recessive acrofacial dysostosis characterised by median mandibular cleft, additional craniofacial anomalies, severe limb defects and learning difficulty, arises not from loss of helicase catalytic activity but from expansion of a 5′ UTR repeat composed of 18-nucleotide and 20-nucleotide motifs; affected individuals carry 14 to 16 repeats compared with 3–12 in unaffected controls, and the expanded allele reduces *EIF4A3* expression. One individual with an atypical presentation carried, in trans with an expanded allele, a missense substitution predicted to disturb the eIF4AIII interface with UPF3B (Favaro et al., 2014). The disorder is therefore a hypomorphic exon junction complex disorder rather than a defect of cap-dependent translation initiation. It illustrates three principles developed in this review: a noncoding variant acting through transcript dosage rather than protein sequence; a quantitative rather than qualitative reduction in a ubiquitously required factor; and a tissue-restricted phenotype, since neural crest derivatives are disproportionately sensitive to reduced eIF4AIII availability (Miller et al., 2017).

### 5.2 RNA editing

RNA editing refers to post-transcriptional modification of RNA sequence, producing nucleotide changes that are not encoded in the underlying DNA. In mammals, the predominant mechanism is adenosine-to-inosine editing, catalysed by adenosine deaminases acting on RNA (ADAR proteins). Inosine is interpreted as guanosine during translation and RNA processing, allowing editing to alter coding potential, splice-site usage, RNA secondary structure, stability and localisation (Slotkin and Nishikura, 2013). A-to-I editing is particularly important in the nervous system, where it fine-tunes transcripts involved in synaptic transmission and neuronal excitability. It also has a critical innate immune function. Because endogenous double-stranded RNA can resemble viral RNA, ADAR-mediated editing helps mark cellular RNA as self. Failure of this process leads to the accumulation of unedited double-stranded RNA and inappropriate activation of interferon signalling.

Loss-of-function variants in *ADAR*, particularly *ADAR1*, are associated with Aicardi-Goutières syndrome, an early-onset encephalopathy characterised by developmental regression, dystonia and elevated expression of interferon-stimulated genes (Fisher and Beal, 2017). Pathogenic variants in *ADAR1* can also cause dyschromatosis symmetrica hereditaria, a pigmentary disorder, reflecting tissue-specific consequences of impaired editing (Suzuki et al., 2005). Thus, RNA editing represents a key regulatory layer that expands transcript diversity and modulates RNA function, while also serving as a critical mechanism for distinguishing self from non-self RNA.

### 5.3 mRNA stability, RNA-Binding proteins, and RNA surveillance

The steady-state level of mRNA within a cell reflects the balance between transcription, processing, stabilisation, and decay. In eukaryotic cells, mRNA degradation typically begins with shortening of the poly(A) tail (deadenylation), which reduces interaction with poly(A)-binding proteins and destabilises the transcript. This is followed by removal of the 5′ cap (decapping), permitting 5′→3′ exonucleolytic degradation, or degradation in the 3′→5′ direction by the exosome complex.

mRNA stability is further modulated by regulatory elements within untranslated regions that are recognised by RNA-binding proteins and microRNAs. RNA-binding proteins (RBPs) play a central role in this process by assembling into ribonucleoprotein complexes that determine transcript fate, coordinating splicing, localisation, translation, and decay. Depending on context, RBPs can either stabilise transcripts or promote their degradation, thereby acting as key determinants of mRNA half-life and translational efficiency (Moore and von Lindern, 2018).

Disruption of RNA-binding protein function is a recognised disease mechanism. Loss of *FMR1* causes Fragile X syndrome through dysregulated translation of synaptic mRNAs, *SMN1* deficiency impairs snRNP assembly and RNA processing in spinal muscular atrophy, and *HNRNPU* variants cause neurodevelopmental disorders through widespread disruption of RNA metabolism (Zhang et al., 2018; Soucek et al., 2019; Alrafiah et al., 2018; Yates et al., 2017; Bramswig et al., 2017).

Additional surveillance pathways extend this quality control system. Nonstop decay targets transcripts lacking a termination codon (Frischmeyer et al., 2002), while no-go decay resolves transcripts with stalled ribosomes (Harigaya and Parker, 2010). Variants that convert a normal stop codon into a sense codon extend translation into the 3′UTR and can destabilise the transcript. A *FOXE3* stop-loss variant (c.959G>T) substitutes the stop codon for a leucine residue and predicts the addition of 72 amino acids to the C-terminus; the mutant allele is often minimally detectable, indicating that the transcript, the protein or both are unstable. The variant is associated with congenital ocular phenotypes (Doucette et al., 2011). Similarly, stop-loss variants in *KISS1R* in Idiopathic hypogonadotropic hypogonadism, show markedly reduced mutant transcript abundance (Seminara et al., 2003; Moalla et al., 2019). Reduced abundance of a stop-loss transcript is compatible with nonstop decay but does not by itself establish it, since canonical nonstop decay requires demonstration that ribosomes translate into the poly(A) tail and that degradation depends on ribosome rescue and exosome function. Neither dependence has been shown directly for these alleles, which are therefore better described as subject to RNA quality control, with nonstop decay, extended 3′ UTR-mediated destabilisation and co-translational degradation of the extended protein remaining plausible and non-exclusive contributors. The distinction is diagnostically material, because a stop-loss variant with reduced transcript on RNA sequencing carries a demonstrated observation but an inferred mechanism.

In no-go decay, ribosome stalling triggers endonucleolytic cleavage of the transcript near the stall site, allowing subsequent exonucleolytic degradation and recycling of ribosomal subunits. These pathways are closely linked to ribosome-associated quality control, which ensures degradation of incomplete polypeptides. Disruption of this system, as seen with biallelic variants in *NEMF*, leads to neuromuscular disease due to the accumulation of aberrant proteins (Martin et al., 2020). This example demonstrates that failure of ribosome rescue and translational surveillance mechanisms can lead to tissue-specific neurodegeneration, highlighting the importance of post-transcriptional quality control at the level of elongating ribosomes.

Together, mRNA stability, RNA-binding proteins and surveillance pathways form an integrated post-transcriptional quality-control network. Disruption of this network alters transcript abundance, translation and proteostasis, linking RNA-level regulation to neurodevelopmental, neuromuscular and multisystem disease.

### 5.4 Noncoding RNAs and post-transcriptional regulation

Noncoding RNAs represent a major regulatory layer in post-transcriptional gene control. Rather than encoding proteins, these transcripts act through interactions with RNA, DNA or proteins to regulate mRNA stability, translation, RNA processing, ribosome function and chromatin state. They include microRNAs, long noncoding RNAs, circular RNAs, small nuclear RNAs and small nucleolar RNAs. Disruption of noncoding RNA genes or their processing pathways can alter developmental gene-expression networks and cause disease despite intact protein-coding sequence.

#### 5.4.1 MicroRNAs (miRNAs)

miRNAs are short (∼22 nucleotide) noncoding RNAs that regulate gene expression by repressing translation and promoting mRNA degradation. They are transcribed as primary miRNA transcripts, processed into precursor hairpins, exported to the cytoplasm and further cleaved to generate mature miRNAs. The mature strand is incorporated into the RNA-induced silencing complex, which recognises target mRNAs, typically through partial complementarity within the 3′ untranslated region (Goel and Goel, 2024). Target recognition depends strongly on the miRNA seed region. Because a single miRNA can regulate many transcripts, and individual transcripts may be regulated by multiple miRNAs, miRNAs form dense post-transcriptional networks that fine-tune development, proliferation and differentiation (Goel and Goel, 2024; Song T. et al., 2026; Savidge et al., 2025; Khoodoruth et al., 2024).

A clinically instructive example is Feingold syndrome, which exists in two genetically distinct forms. Type 1 results from haploinsufficiency of *MYCN*, a transcription factor. Type 2 is caused by loss-of-function variants affecting the miR-17-92 cluster encoded by *MIR17HG*. Although both forms share overlapping phenotypes, functional studies demonstrate distinct mechanisms. Loss of the miRNA cluster leads to dysregulation of TGF-β signalling, whereas the transcription factor-related form (*MYCN*) primarily affects PI3K signalling. This illustrates that similar phenotypes can arise from the disruption of distinct regulatory layers, including post-transcriptional control (Mirzamohammadi et al., 2018).

#### 5.4.2 Long noncoding RNAs (lncRNAs)

lncRNAs are transcripts longer than 200 nucleotides that lack protein-coding capacity but regulate gene expression through diverse molecular mechanisms. They can influence chromatin state, transcription, RNA stability and RNA-binding protein activity. Some act locally in cis to regulate neighbouring genes, while others function at distant loci. Disruption of lncRNAs can cause Mendelian disease by altering developmental gene dosage without changing protein-coding sequence. Homozygous deletions of *LINC01956* were shown to cause a severe congenital limb and hindbrain malformation syndrome through loss of target gene expression of *EN1* (Allou et al., 2021)*.* A particularly instructive example involves *CHASERR*, an lncRNA located adjacent to *CHD2*. Deletions affecting *CHASERR*, while leaving *CHD2* intact, result in increased *CHD2* expression in cis and lead to severe neurodevelopmental phenotypes. This demonstrates that lncRNAs can act as local transcriptional regulators and that their disruption can alter gene dosage independently of coding sequence variation (Ganesh et al., 2024). These examples illustrate that disruption of lncRNA expression, whether by deletion, duplication, or chromosomal rearrangement, can alter the dosage of key developmental genes and cause Mendelian disorders, even when coding sequences remain intact.

#### 5.4.3 Circular RNAs (circRNAs)

Circular RNAs (circRNAs) are generated by back-splicing, in which a downstream splice donor is joined to an upstream splice acceptor. Unlike linear mRNAs, circRNAs lack 5′ caps and 3′ poly(A) tails, making them resistant to exonuclease-mediated degradation and thus relatively stable within cells They are enriched in the nervous system, show developmental regulation and are abundant at synapses (Amelimojarad and Amelimojarad, 2025; Song and Kim, 2025; Song Z. et al., 2026). CircRNAs can regulate gene expression by interacting with microRNAs, RNA-binding proteins and, in some cases, the transcriptional machinery of their parental genes. Their biogenesis depends on RNA structure, splice-site selection and RNA-binding protein activity. ADAR-mediated editing of double-stranded RNA structures can influence circularisation, while spliceosomal defects may alter back-splicing efficiency. No monogenic disorder has yet been definitively attributed to primary circRNA dysfunction. However, Mendelian disorders affecting RNA editing, splicing or RNA-binding proteins can secondarily perturb circRNA biogenesis. For example, loss of ADAR function alters RNA editing and RNA structure, while variants in spliceosomal components such as SF3B1 or snRNP-associated pathways can change circRNA profiles by disrupting splice-site recognition. These observations suggest that circRNAs form part of the broader RNA-regulatory network affected in neurodevelopmental and neurological disease, even though their primary causal role remains less well established (Uguen et al., 2026).

#### 5.4.4 Small nuclear RNAs (snRNAs)

Small nuclear RNAs (snRNAs) are essential RNA components of the spliceosome. Each major spliceosomal snRNA class is encoded by a set of paralogous genes, some of which are independently susceptible to pathogenic variation. The canonical U2-1 snRNA is encoded by the multicopy *RNU2-1* locus, whereas the single-copy paralog *RNU2-2* encodes the distinct U2-2 snRNA. U2-2 is expressed and incorporated into the major spliceosome, and pathogenic *RNU2-2* variants cause clinically distinct neurodevelopmental disorders. U2 snRNA base-pairs with the intronic branch-point region and subsequently interacts with U6 to contribute to formation of the catalytic spliceosomal core. Similarly, *RNU4-2* encodes U4-2 snRNA, one of the U4 paralogs incorporated into the U4/U6·U5 tri-snRNP of the major spliceosome. Before catalysis, U4 forms an extensive duplex with U6 and maintains U6 in a non-catalytic configuration. During spliceosome activation, helicase-mediated remodelling unwinds the U4/U6 duplex and releases U4, allowing U6 to interact with U2 and the 5′ splice site to form the catalytic core. Recurrent pathogenic *RNU4-2* variants cluster within structurally constrained regions of U4-2 involved in U4/U6 interaction and tri-snRNP activation. Rather than disrupting a single target transcript, these variants produce biased, transcriptome-wide mis-splicing, with vulnerable splice sites showing exon skipping, intron retention or cryptic splice-site usage. This distributed molecular effect is consistent with the severe neurodevelopmental phenotype and explains why bulk RNA analysis may reveal a broad splicing signature rather than one pathognomonic aberrant isoform (Chen et al., 2024; Jackson et al., 2025; Greene et al., 2025) (Figure 3).

**FIGURE 3:**
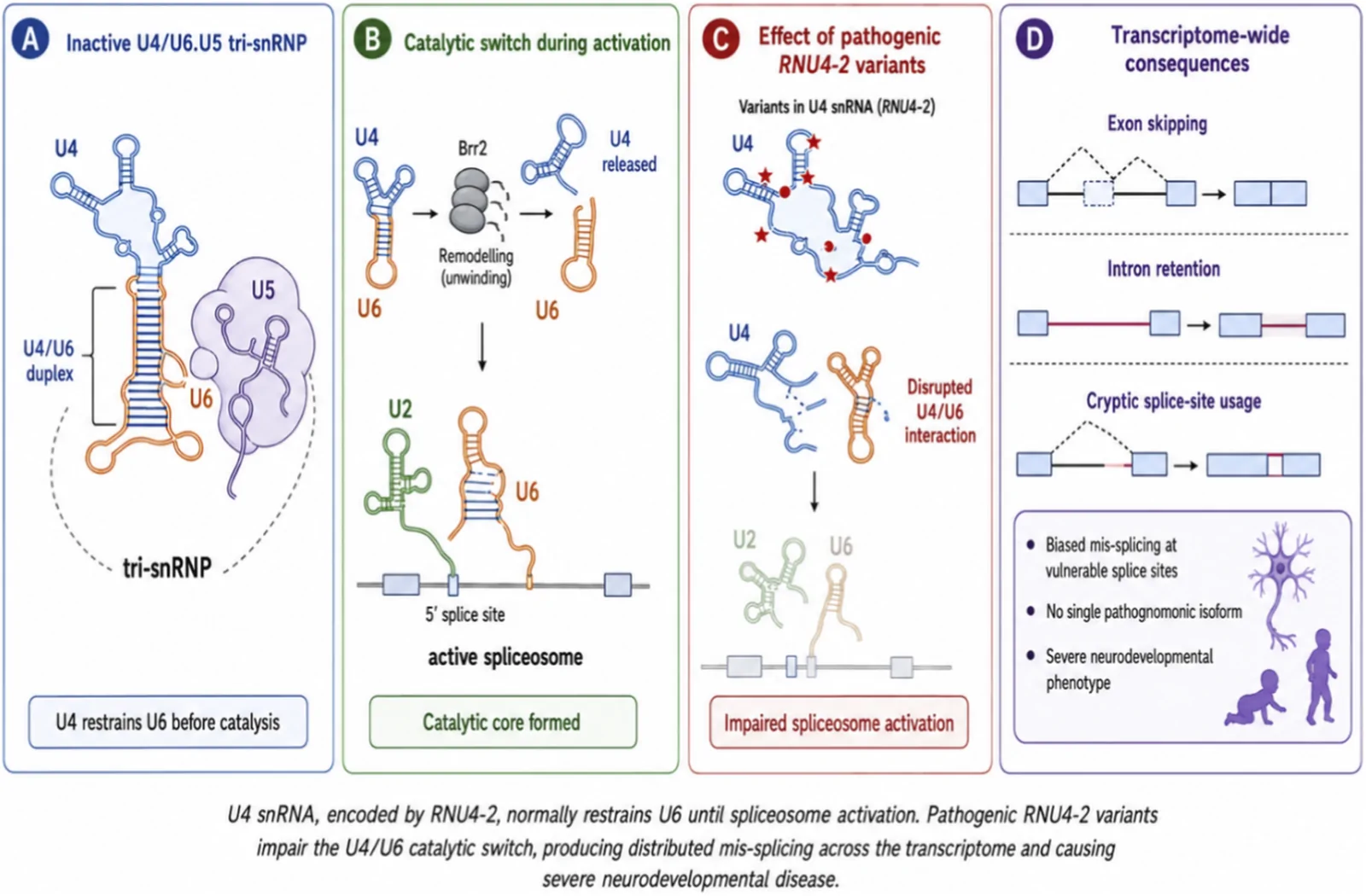
Pathogenic *RNU4-2* variants disrupt the U4/U6 catalytic switch and cause transcriptome-wide mis-splicing. *RNU4-2* encodes U4-2 snRNA, a component of the U4/U6·U5 tri-snRNP. **(A)** U4 base-pairs with U6 and restrains it before catalysis. **(B)** Spliceosome activation releases U4, allowing U6 to interact with U2 and the 5′ splice site to form the catalytic core. **(C)** Pathogenic *RNU4-2* variants disrupt U4/U6 interaction and tri-snRNP remodelling. **(D)** The result is biased transcriptome-wide mis-splicing, including exon skipping, intron retention and cryptic splice-site use, underlying RNU4-2-related neurodevelopmental disease.

#### 5.4.5 Small nucleolar RNAs (snoRNAs)

SnoRNAs, 60-300 nt, are nuclear noncoding RNAs that guide chemical modification of ribosomal RNA, small nuclear RNA and other RNA species. Their primary role is to ensure correct maturation and structural stability of the ribosome, which is essential for accurate protein synthesis. Two major classes of snoRNAs are defined by their structure and function. C/D box snoRNAs (SNORD family) guide 2′-O-methylation of ribose sugars, a modification that stabilises RNA structure, protects against degradation and supports proper ribosome assembly. H/ACA box snoRNAs (SNORA family) guide pseudouridylation, in which uridine is converted to pseudouridine, enhancing hydrogen bonding capacity and improving RNA structural stability.

Disruption of snoRNA function can lead to human disease. Biallelic variants in *SNORD118* impair ribosomal RNA processing and cause leukoencephalopathy with calcifications and cysts, characterised by progressive neurological decline and distinctive neuroimaging findings (Jenkinson et al., 2016). Variants affecting *SNORA31* have been associated with susceptibility to herpes simplex encephalitis, reflecting impaired RNA modification and altered innate immune responses (Lafaille et al., 2019). Through their roles in RNA modification, snoRNAs influence ribosome assembly, translation accuracy and cellular homeostasis. Their dysfunction demonstrates that defects in RNA processing can affect global protein synthesis and contribute to neurodevelopmental and neurological disease.

### 5.5 mRNA localisation

Following processing and export, mRNAs are not translated uniformly throughout the cell. Instead, their localisation and translational efficiency are tightly regulated, enabling spatial and temporal control of protein synthesis. This is particularly critical in highly polarised cells such as neurons, where local translation at dendrites and synapses supports rapid and activity-dependent responses. mRNA localisation is directed by cis-acting sequence elements, typically within the 3′ untranslated region (3′ UTR), often termed “zip codes”. These motifs are recognised by RNA-binding proteins that assemble messenger ribonucleoprotein (mRNP) complexes. These interactions direct transcripts to specific subcellular compartments via cytoskeletal transport and localise them within ribonucleoprotein granules. Translation is frequently repressed during transport and activated only upon arrival at the target site, allowing precise coupling of localisation and protein synthesis (Wang J. et al., 2023).

Disruption of mRNA localisation is associated with human disease. In *PABPN1*-related oculopharyngeal muscular dystrophy, a short GCG repeat expansion in *PABPN1* alters nuclear RNA processing and leads to intranuclear aggregates that sequester polyadenylated RNAs. This impairs RNA export, localisation and downstream protein synthesis, contributing to progressive myofibre dysfunction (Mezzell et al., 2026).

mRNA localisation represents a distinct post-transcriptional regulatory layer that links RNA transport to cellular architecture. Disruption of this process alters spatial gene expression and contributes to disease, particularly in highly specialised cells. Some exported mRNAs are also maintained in a translation-repressed but activation-competent state within ribonucleoprotein granules, stress granules or processing bodies. These structures provide reversible RNA storage, allowing rapid cellular responses without new transcription. Disruption of RNA storage and granule dynamics, including through variants in *FUS* and *TARDBP*, causes abnormal RNA metabolism and contributes to neurodegeneration (Kim et al., 2021).

Post-transcriptional regulation governs RNA fate through coordinated control of processing, stability, surveillance, localisation and storage. These mechanisms act as interconnected checkpoints that refine gene expression beyond transcription. Together, they balance diversity (e.g., alternative splicing, RNA editing) with precision (e.g., RNA quality control and stability). Disruption of these processes alters RNA integrity, abundance or spatial distribution, providing a major mechanism of disease despite intact transcription.

## 6 Translational regulation

Translational regulation determines whether, when and how efficiently an mRNA is converted into protein. It provides a critical layer of control downstream of transcription and RNA processing, allowing cells to rapidly adjust protein output in response to developmental signals, environmental cues and cellular stress. Eukaryotic translation proceeds through initiation, elongation, termination and ribosome recycling. Initiation is the principal regulatory checkpoint, involving recognition of the mRNA, recruitment of the ribosomal pre-initiation complex, start codon selection and assembly of the functional ribosome. Elongation, termination and ribosome rescue also influence translational fidelity, protein folding, ribosome traffic and quality control.

Because translation determines functional protein abundance, pathogenic variants can cause disease by altering translation efficiency, codon decoding, stop-codon recognition, ribosome dynamics or translational surveillance, even when mRNA abundance is unchanged. Translational regulation therefore forms a dynamic interface between transcript availability and proteomic output.

### 6.1 Regulation of translation initiation

#### 6.1.1 Cap-dependent initiation and mTOR signalling pathway

Translation initiation is the major rate-limiting and regulatory step in protein synthesis. During cap-dependent initiation, the 5′ cap is recognised by eIF4E, which assembles with other initiation factors to recruit the ribosomal pre-initiation complex. The ribosome then scans the 5′ UTR to identify the start codon, allowing assembly of the complete ribosome and initiation of protein synthesis. Disruption of translation initiation causes human disease. Variants in *EIF2B1* to *EIF2B5* cause Vanishing White Matter disease (Shivaram et al., 2022). The five subunits assemble into the decameric eIF2B complex, the guanine nucleotide exchange factor that regenerates active eIF2-GTP from eIF2-GDP after each round of initiation. Pathogenic variants reduce this exchange activity and lower the availability of the eIF2-GTP-Met-tRNAi ternary complex, constraining initiation and leaving the integrated stress response inappropriately sustained (Shivaram et al., 2022). Because phosphorylated eIF2α acts as a competitive inhibitor of eIF2B, stress-induced eIF2α phosphorylation and germline loss of eIF2B function converge on the same regulatory node, which is why Vanishing White Matter disease appears both here and in the discussion of the integrated stress response below. Similarly, pathogenic variants in *EIF2S3* lead to MEHMO syndrome, reflecting defective initiation complex formation (Skopkova et al., 2017).

Translational control is further modulated by cellular signalling pathways. The mTOR pathway integrates nutrient, growth-factor and energy signals to regulate protein synthesis, primarily by controlling the availability of eIF4E. Dysregulation of this pathway underlies disorders such as tuberous sclerosis complex and *PTEN*-related syndromes, linking aberrant translation to neurodevelopmental disease and tumourigenesis. Smith-Kingsmore syndrome (gain-of-function *MTOR* variants) presents with macrocephaly, developmental delay and seizures (Besterman et al., 2021).

Under cellular stress, phosphorylation of eIF2α reduces global translation while permitting selective translation of stress-response transcripts such as *ATF4*. This adaptive response is essential for cellular homeostasis, and its disruption contributes to disorders such as Vanishing White Matter disease and Wolcott-Rallison syndrome (Delepine et al., 2000).

In addition to cap-dependent initiation, a subset of mRNAs can be translated via cap-independent mechanisms, including internal ribosome entry sites. These pathways become particularly important under stress conditions and can contribute to disease when aberrantly activated, as demonstrated in repeat expansion disorders.

#### 6.1.2 Cap-independent translation internal ribosome entry sites (IRES)

IRES are structured RNA elements located within the 5′ UTRs of specific mRNAs that permit ribosome recruitment independently of the 5′ 7-methylguanosine cap and eIF4E. This allows selective protein synthesis under conditions in which global translation is suppressed, such as cellular stress, hypoxia or viral infection. This mechanism is particularly important for maintaining expression of specific transcripts during stress responses, and cap-independent translation can also generate alternative protein products from the same transcript.

*CACNA1A* normally encodes a voltage-gated calcium channel; however, the same mRNA can also generate a second protein, α1ACT, through internal ribosome entry site (IRES)-mediated translation initiating at an alternative internal site. α1ACT functions as a transcription factor that coordinates a programme of genes required for neural and Purkinje cell development and polyglutamine expansion of α1ACT underlies spinocerebellar ataxia type 6, illustrating how a cap-independent product can itself be a disease determinant (Marques et al., 2022; Du et al., 2013). Dysregulation of IRES-mediated translation can also contribute to disease through selective effects on specialised initiation. Diamond-Blackfan anaemia is mainly caused by haploinsufficiency of *RPS19* and *RPL11*, which impairs ribosome function and selectively disrupts translation of transcripts required for erythropoiesis, including those dependent on specialised initiation mechanisms (Khajuria et al., 2018). Thus, IRES-mediated translation represents an adaptive mechanism that ensures selective protein synthesis under stress, but when dysregulated can contribute to disease through aberrant or ectopic protein production.

#### 6.1.3 Upstream open reading frames and translational control

Upstream open reading frames (uORFs) are short coding sequences located within the 5′ UTRs that regulate translation of the main coding sequence. Ribosomes typically initiate translation at uORFs before reaching the primary start codon, thereby reducing or modulating downstream protein synthesis. uORFs therefore act as cis-regulatory elements that control translation efficiency in a transcript- and context-dependent manner.

In hereditary thrombocythemia, variants in *THPO* disrupt an inhibitory uORF, leading to increased translation and excessive platelet production (Kimura et al., 2024). Conversely, variants that create or strengthen inhibitory uORFs can reduce translation of the main protein product. Cornelia de Lange syndrome (CdLS) is characterised by intellectual disability, distinctive facial features, growth retardation, feeding difficulties and multiple organ system abnormalities. *NIPBL* variants occur in approximately 80% of CdLS cases, and pathogenic variants in the 5′ UTR of *NIPBL* (c.-467C>T, c.-321-320delCCinsA, c.-94C>T) have been identified in patients with CdLS (Chen et al., 2022; Borck et al., 2006). This highlights the importance of 5′ UTRs as critical regulatory elements in gene expression and disease (Scheper et al., 2006).

Recent genome-wide analyses have reinforced the clinical importance of 5′ UTR variation. Machine-learning approaches such as 5ULTRA show that variants affecting Kozak sequence strength, uORF creation, uORF disruption, uORF overlap or uORF stop codons are enriched in disease-associated genes and can substantially alter protein output. These findings provide a mechanistic basis for disease caused by altered ribosome scanning, initiation and reinitiation dynamics, independent of coding-sequence change (Chaldebas et al., 2026).

Ribosomes are not necessarily functionally interchangeable. Variation in ribosomal-protein composition, rRNA modification, assembly state and associated factors can alter the translation of particular transcript classes, especially mRNAs with structured 5′ UTRs, upstream open reading frames or stringent initiation requirements. Evidence for specialised translation is strongest in developmental and stress contexts, but interpretation requires caution because apparent selectivity may also arise from altered ribosome abundance, cell composition or secondary stress responses. Ribosomopathies demonstrate the clinical principle: a broadly expressed defect in ribosome biogenesis can produce tissue-selective phenotypes because progenitor populations differ in translational demand, p53 sensitivity and dependence on specific transcript programmes. Functional studies should therefore distinguish global reduction in protein synthesis from selective changes in ribosome occupancy.

### 6.2 Regulation of elongation and ribosome dynamics

Although translation initiation is the principal regulatory step, elongation is also subject to precise control and contributes to overall protein output and fidelity.

#### 6.2.1 Elongation factor modulation

Elongation determines the speed and fidelity of codon decoding, influences ribosome traffic and affects co-translational protein folding. Variants that alter elongation factors, codon optimality, synonymous regulatory elements, stop-codon context or ribosome rescue pathways can therefore modify protein abundance or quality without necessarily changing mRNA levels.

During elongation, aminoacyl-tRNAs are delivered to the ribosome by the eEF1 complex. eEF1A2 binds and delivers charged tRNAs in a GTP-dependent manner, while the eEF1B complex recycles eEF1A to sustain elongation efficiency. Ribosomal translocation is mediated by eEF2, which moves the ribosome along the mRNA after peptide-bond formation. Disruption of these factors is particularly relevant in neurons, where precise protein synthesis is required for synaptic development and function. Pathogenic variants in *EEF1A2* cause epilepsy, intellectual disability and autism, and variants in the core elongation machinery, comprising the eEF1B complex subunits *EEF1B2* and *EEF1D* and the translocase *EEF2* are associated with neurodevelopmental disorders characterised by developmental delay, microcephaly, seizures or behavioural abnormalities (Carvill et al., 2020; Hekman et al., 2012; Nabais Sa et al., 2021; Prasun and Patra, 2024). *VARS1* (formerly *VARS*) encodes cytoplasmic valyl-tRNA synthetase and acts upstream of elongation by charging tRNA-Val rather than functioning as an elongation factor; biallelic variants cause a developmental and epileptic encephalopathy with microcephaly (Aynekin et al., 2026). Aminoacyl-tRNA synthetase disorders are considered alongside elongation-factor disorders because they constrain elongation through substrate supply, but the biochemical lesion is distinct and the two groups should not be conflated.

Synonymous variants can also disrupt translation despite preserving the amino acid sequence. They may alter codon optimality, local mRNA structure, ribosome pausing, co-translational folding, splicing regulatory elements, RNA-binding protein sites or microRNA-binding sites. Codons matched to abundant tRNAs are generally translated efficiently, whereas rare or context-dependent codons can slow elongation and alter protein folding or stability. Thus, synonymous substitutions may act through translational, post-transcriptional or RNA-structural mechanisms rather than through primary protein-sequence change.

Codon bias, the non-random use of synonymous codons across genomes and local sequence contexts, links these effects to elongation: codons matched to abundant tRNAs are decoded efficiently, whereas rare codons slow elongation and can disrupt co-translational folding, domain assembly and protein stability.

Translation termination is typically mediated by eukaryotic release factors (eRF1 and eRF3), which recognise stop codons and promote polypeptide release. Termination fidelity depends on stop-codon identity and sequence context. UGA is generally the most permissive stop codon for readthrough, whereas UAA is the most stringent; a pyrimidine at the +4 position can increase readthrough, while guanine promotes efficient termination. In some contexts, near-cognate tRNAs compete with release factors, allowing stop-codon readthrough. Clinically, readthrough may partially rescue premature termination codons, but it can also generate abnormal C-terminally extended proteins. Pharmacological readthrough has been explored in disorders such as Duchenne muscular dystrophy and cystic fibrosis (Roberts et al., 2023; Buck et al., 2009).

A specialised elongation-related mechanism is eIF5A hypusination. eIF5A is activated by a unique post-translational modification in which DHPS converts a lysine residue to deoxyhypusine and DOHH converts this intermediate to hypusine. Hypusinated eIF5A facilitates elongation across difficult sequence contexts, particularly polyproline tracts and other motifs that promote ribosome stalling. Impaired hypusination causes selective translational defects rather than uniform suppression of protein synthesis, disproportionately affecting proteins involved in cytoskeletal organisation, signalling and neuronal function. Pathogenic variants in *DHPS*, *DOHH* and *EIF5A* are associated with neurodevelopmental disorders characterised by developmental delay, intellectual disability, seizures and microcephaly (Shojaeinia et al., 2024; Ziegler et al., 2022; Park et al., 2022; Guo et al., 2023; Barba-Aliaga and Alepuz, 2022). This pathway links elongation control to ribosome-associated quality control and proteostasis.

#### 6.2.2 Ribosome stalling and quality control

Ribosome stalling can occur during elongation when ribosomes encounter damaged RNA, secondary structures, premature polyadenylation or difficult-to-translate sequence contexts such as polybasic or proline-rich regions (Chang and Choe, 2026). Transcripts lacking a normal stop codon may cause ribosomes to translate into the poly(A) tail, generating polylysine tracts that stall elongation. Proline-rich sequences can also slow peptide-bond formation because proline is structurally constrained and relatively inefficient as both a donor and acceptor during peptide-bond synthesis.

Cells have evolved ribosome-associated quality control (RQC) mechanisms to detect and resolve stalled ribosomes. Key components include ZNF598, which senses ribosome collisions and initiates ubiquitination of ribosomal proteins; NEMF, which mediates processing of stalled nascent chains; LTN1, an E3 ubiquitin ligase that targets incomplete polypeptides for degradation; and ANKZF1, which facilitates release of stalled nascent chains. Together, these factors promote disassembly of stalled ribosomes, degradation of aberrant peptides and recycling of translational machinery. Biallelic variants in *NEMF* cause a neuromuscular disorder through accumulation of aberrant, incompletely degraded nascent proteins, demonstrating that failure of nascent-chain quality control can produce tissue-selective neurodegeneration (Martin et al., 2020) (Figure 4).

**FIGURE 4:**
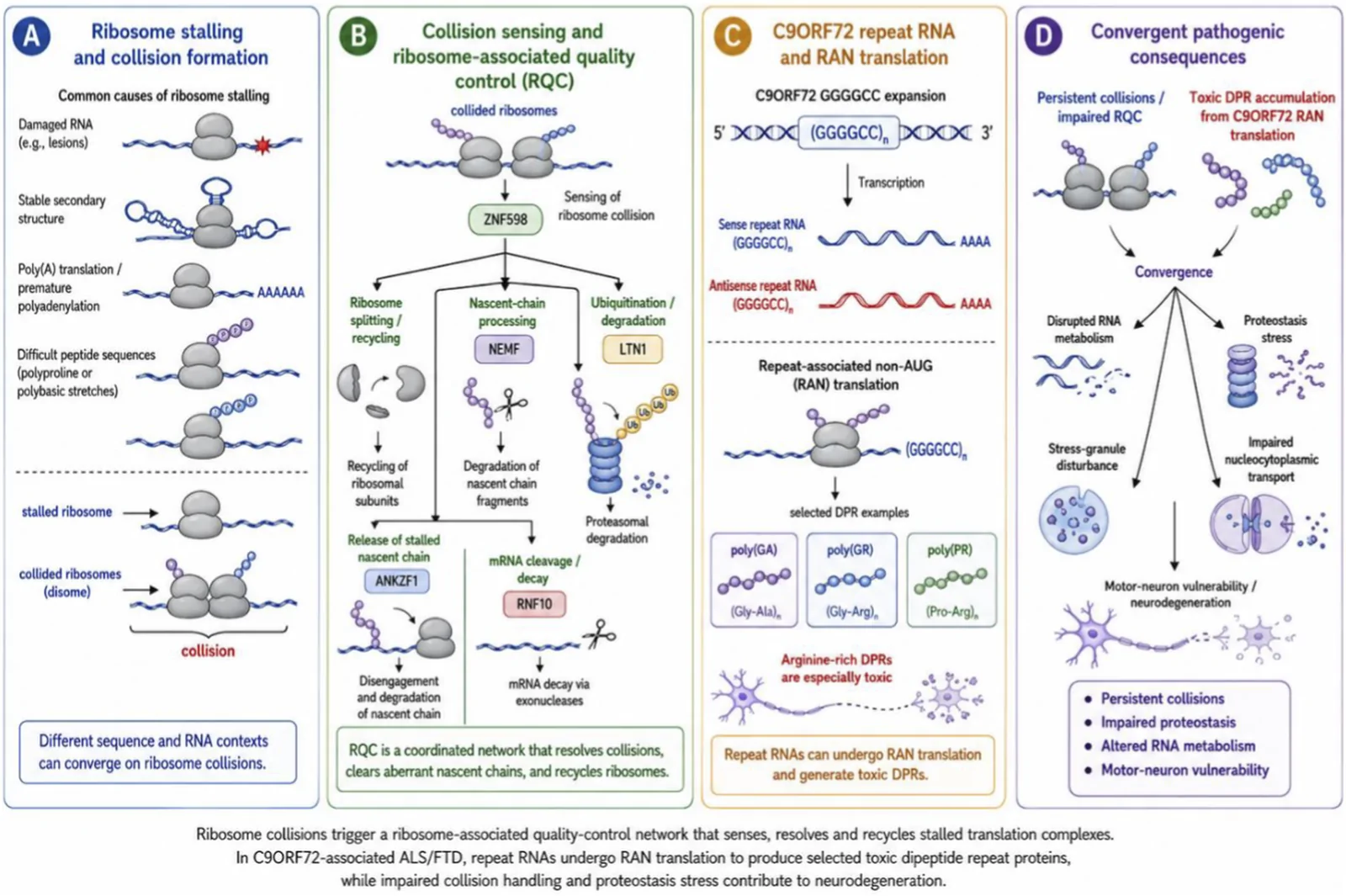
Ribosome collision sensing, ribosome-associated quality control and *C9ORF72*-associated RAN translation. **(A)** RNA damage, secondary structures, premature polyadenylation and difficult peptide sequences cause ribosome stalling and collisions. **(B)** ZNF598 initiates ribosome-associated quality control, including ribosome recycling, nascent-chain processing, LTN1-mediated degradation and mRNA decay. **(C)** Expanded *C9ORF72* repeat RNAs undergo RAN translation, producing toxic dipeptide repeat proteins; selected examples are shown. **(D)** Persistent collisions and toxic DPR accumulation disrupt RNA metabolism, proteostasis, stress granules and nucleocytoplasmic transport, promoting motor-neuron degeneration.

Failure of RQC is also implicated in neurodegeneration through aberrant translation products. A pathogenic expansion of a GGGGCC (G4C2) hexanucleotide repeat within the first intron of *C9ORF72* is the most common genetic cause of amyotrophic lateral sclerosis and frontotemporal dementia. These transcripts can undergo repeat-associated non-AUG translation, producing toxic dipeptide repeat proteins, particularly arginine-rich species such as poly(glycine-arginine) and poly(proline-arginine), which disrupt ribosome function, RNA metabolism and cellular proteostasis. In *C9ORF72*-associated disease, impaired activity of quality-control components, including ZNF598, may reduce the ability to clear stalled translation complexes. Persistent aberrant translation and accumulation of toxic dipeptide repeat proteins contribute to motor-neuron vulnerability and progressive neurodegeneration (Zu et al., 2013).

### 6.3 Translational control in development and differentiation

Translational regulation is essential during development because it allows rapid, selective control of protein synthesis independent of transcription. During early embryogenesis, when zygotic transcription is limited, development depends heavily on maternally deposited mRNAs that are stored in a translationally repressed state and activated in a precise temporal sequence after fertilisation.

In stem and progenitor cells, many differentiation-related transcripts are already present but remain translationally silenced until developmental cues trigger their selective activation. This enables rapid and coordinated changes in protein expression during lineage commitment. In differentiated tissues, translational control becomes spatially specialised: neurons transport mRNAs to dendrites and axons, where local translation supports synaptic plasticity, axonal guidance and activity-dependent responses.

Disruption of translational capacity during development is exemplified by pathogenic variants in *TCOF1*. *TCOF1* encodes a nucleolar protein required for ribosomal RNA transcription and ribosome biogenesis. Impaired ribosome production reduces global protein synthesis and activates cellular stress pathways, particularly in neural crest cells, which have high translational demand during craniofacial development. Selective neural crest vulnerability leads to the characteristic craniofacial abnormalities of Treacher Collins syndrome (Wang F. et al., 2023).

Collectively, translational dysregulation may result in altered protein abundance, defective protein structure or selective vulnerability of specific cell types. Neurons are particularly susceptible owing to their high metabolic demand and reliance on tightly regulated, spatially restricted protein synthesis. Thus, translational regulation integrates cellular state with proteomic output, acting alongside transcriptional and post-transcriptional mechanisms to ensure precise control of gene expression. Disruption of this balance represents a central mechanism underlying a broad spectrum of human disease.

## 7 Post-translational regulation

Post-translational regulation governs protein function after synthesis. It determines whether a protein is correctly folded, modified, localised, activated, incorporated into complexes or degraded. This layer therefore shapes cellular function independently of mRNA abundance or translation rate and integrates multiple processes, including protein folding, covalent modification, trafficking and degradation, which together maintain proteostasis and enable dynamic cellular responses.

Major post-translational mechanisms include protein folding and quality control, covalent post-translational modifications, intracellular trafficking and regulated protein degradation. Together, these processes maintain proteostasis and allow rapid cellular responses to developmental signals, metabolic state and environmental stress.

Disruption of post-translational regulation can cause disease by reducing functional protein abundance, altering protein activity, impairing localisation or allowing toxic protein accumulation. These mechanisms are central to many metabolic, neurodevelopmental, neurodegenerative and multisystem disorders.

### 7.1 Protein folding and quality control

Newly synthesised polypeptides must achieve precise three-dimensional conformations to become functionally active. Protein folding begins co-translationally at the ribosome and continues within the cytosol or endoplasmic reticulum (ER), assisted by molecular chaperones, including heat shock proteins such as HSP70 and HSP90. These chaperones stabilise nascent polypeptides, prevent aggregation and facilitate correct folding through ATP-dependent cycles.

Misfolded proteins are selectively targeted for degradation through coordinated quality control systems. The primary pathway is ER-associated degradation (ERAD), which recognises misfolded proteins, retrotranslocates them to the cytosol and targets them for proteasomal degradation. Aggregation-prone proteins are cleared via the autophagy-lysosome pathway.

Disruption of protein folding is a well-established disease mechanism. In cystic fibrosis, variants in *CFTR*, most commonly p.Phe508del, result in misfolding and retention within the ER, followed by degradation, leading to loss of functional protein at the cell surface (Trouve and Ferec, 2025). Similarly, variants affecting chaperone systems, including *HSPB1* and *HSPB8*, cause peripheral neuropathies through impaired proteostasis and accumulation of misfolded or aggregation-prone proteins (Almeida-Souza et al., 2010; Irobi et al., 2012).

### 7.2 Post-translational modifications (PTMs)

Post-translational modifications are covalent chemical changes that regulate protein activity, stability, localisation, and interactions. Multiple PTMs often act in combination to generate complex regulatory outputs. Major PTMs include phosphorylation, ubiquitination, acetylation, methylation, glycosylation, lipidation, SUMOylation, ADP-ribosylation and proteolytic processing. Disruption of these pathways can cause disease by altering signalling, chromatin regulation, protein trafficking, proteostasis or cellular metabolism (Table 3).

**TABLE 3:** Major post-translational modifications relevant to Mendelian disease.

Modification	Writers/erasers	Major acceptor residue(s)	Principal functional effects	Selected mendelian relevance
Phosphorylation	Protein kinases; serine/threonine and tyrosine phosphatases	Serine, threonine, tyrosine	Rapid regulation of enzyme activity, signalling, localisation, protein interactions, cell-cycle progression and degradation	*CSNK2A1*-related Okur–Chung syndrome; *PPP2R5D*-related neurodevelopmental disorder; kinase-pathway developmental syndromes
Ubiquitination	E1 activating enzymes, E2 conjugating enzymes, E3 ligases; deubiquitinases	Predominantly lysine; occasionally N-terminus or non-lysine sites	Proteasomal degradation, endocytosis, DNA repair, chromatin regulation, signalling and stress responses; outcome depends on chain linkage	*UBE3A*-related Angelman syndrome; *TRIP12*-related disorder; *HUWE1*-related neurodevelopmental disorder
Acetylation	Lysine acetyltransferases; histone deacetylases and sirtuins	Lysine and protein N-termini	Neutralises lysine charge; regulates chromatin accessibility, transcription, protein stability, interactions and metabolism	*CREBBP/EP300*-related Rubinstein–Taybi syndrome; *HDAC8*-related Cornelia de Lange spectrum
Methylation	Lysine, arginine or histidine methyltransferases; demethylases	Lysine, arginine and histidine	Context-dependent regulation of chromatin, transcription, RNA processing, DNA repair and protein interactions	*KMT2D*-related Kabuki syndrome; *EHMT1*-related Kleefstra syndrome; *PRMT7*-related developmental disorder
Glycosylation	Glycosyltransferases; glycosidases; ER/Golgi processing enzymes	Asparagine (N-linked); serine/threonine (O-linked); selected other residues	Protein folding, quality control, stability, trafficking, cell–cell recognition and receptor function	Congenital disorders of glycosylation, including *ALG1-CDG*; O-*GlcNAc*-cycle disorders
Lipidation: S-palmitoylation	ZDHHC palmitoyltransferases; acyl-protein thioesterases	Cysteine	Reversible membrane association, trafficking, signalling and protein-complex assembly	*ZDHHC9*-related X-linked intellectual disability; emerging palmitoylation-pathway disorders
Lipidation: prenylation	Farnesyltransferase or geranylgeranyltransferases; CAAX-processing enzymes	C-terminal cysteine within a CAAX motif	Irreversible hydrophobic modification supporting membrane targeting and signalling	Rasopathy-relevant pathway biology; *ICMT*- and prenylation-pathway developmental disorders
Lipidation: N-myristoylation	N-myristoyltransferases	N-terminal glycine after initiator methionine removal	Co-translational or post-translational membrane targeting, conformational switching and complex assembly	*NMT1*-related neurodevelopmental phenotypes and infectious-disease biology
ADP-ribosylation	PARP/ART enzymes; PARG, ARH1 and ARH3 hydrolases	Serine is prominent in the DNA-damage response; glutamate, aspartate, lysine, arginine and other residues may also be modified	DNA-damage signalling, chromatin relaxation, transcription, stress responses, cell death and metabolism	*HPF1*–*PARP1*-dependent serine ADP-ribosylation is central to DNA repair; pathway defects are relevant to neurodegeneration and genome-instability phenotypes
SUMOylation	SAE1/SAE2 E1, UBC9 E2, SUMO E3 ligases; SENP proteases	Lysine, often within a Ψ-K-x-E motif	Nuclear transport, transcription, chromatin regulation, genome stability, protein localisation and crosstalk with ubiquitination	SUMO-pathway disruption is implicated in developmental and neurological disorders; gene-specific Mendelian associations continue to expand

#### 7.2.1 Phosphorylation

Phosphorylation is a reversible post-translational modification in which protein kinases transfer a phosphate group from ATP to serine, threonine or tyrosine residues. Protein phosphatases reverse this process. Phosphorylation regulates enzyme activity, protein interactions, localisation, stability and signal transduction.

Signal transduction pathways frequently depend on phosphorylation cascades. The MAPK/ERK pathway and the PI3K-AKT-mTOR pathways are major examples, regulating cell growth, proliferation, differentiation and survival. This hierarchical structure ensures signal specificity and amplification while allowing integration with other signalling networks. Germline variants affecting phosphorylation-dependent signalling pathways, including RAS-MAPK and mTOR-related pathways, underlie multiple Mendelian developmental disorders (Nishio et al., 2023; Kato et al., 2019). Activating variants in genes encoding phosphatidylinositol 3-kinase (PI3K)-AKT pathway components cause megalencephaly-polymicrogyria-polydactyly-hydrocephalus syndrome. Individuals with MPPH lacking upstream PI3K-AKT pathway variants carry *de novo* variants in *CCND2* (encoding cyclin D2) that cluster around a residue phosphorylated by glycogen synthase kinase 3β (GSK-3β). These variants render cyclin D2 resistant to proteasomal degradation, increasing protein stability and disrupting neurodevelopmental growth control (Mirzaa et al., 2014).

#### 7.2.2 Ubiquitination and deubiquitination

Ubiquitination is the covalent attachment of ubiquitin to lysine residues on target proteins (Sabeh et al., 2025; Bossuyt et al., 2021; Jiang and Beaudet, 2004). It is mediated by E1 activating enzymes, E2 conjugating enzymes and E3 ubiquitin ligases, and reversed by deubiquitinating enzymes. Ubiquitination tags proteins for degradation via the proteasome and also regulates protein localisation, interactions and signalling.

Deubiquitination is carried out by deubiquitinating enzymes (DUBs), which cleave ubiquitin from substrate proteins or disassemble ubiquitin chains. By removing ubiquitin, DUBs can rescue proteins from degradation, edit ubiquitin chain length or linkage type, and recycle ubiquitin for reuse in the cell. Variants in deubiquitinating enzymes such as *STAMBP* and *USP9X* result in neurodevelopmental disorders. Biallelic *STAMBP* variants cause MIC-CAP syndrome, with microcephaly, developmental delay and vascular malformations, as impaired receptor signalling in neural progenitors affects gene expression programmes critical for neurodevelopment and vascular patterning (Hu et al., 2022). Variants in *USP9X* cause neurodevelopmental disorders through altered protein turnover (Reijnders et al., 2016).

#### 7.2.3 Acetylation and methylation

Acetylation, mediated by histone acetyltransferases and reversed by deacetylases, regulates chromatin accessibility and transcription. Variants in *CREBBP* and *EP300* cause Rubinstein–Taybi syndrome, illustrating the role of acetylation in development (Bartsch et al., 2002).

Protein methylation, most commonly on lysine or arginine residues, is mediated by methyltransferases and reversed by demethylases. Its effects are highly context-dependent. Histone methylation regulates chromatin structure and transcriptional activation or repression, whereas methylation of non-histone proteins can alter transcription-factor activity, protein-protein interactions, signal transduction, DNA repair and protein stability (Giaimo et al., 2024). Thus, methylation functions both as a chromatin-regulatory mark and as a broader post-translational mechanism controlling protein behaviour and cellular state.

#### 7.2.4 Additional modifications

Additional post-translational modifications further expand protein-level regulation. SUMOylation is the covalent attachment of small ubiquitin-like modifier (SUMO) proteins to lysine residues on target proteins. Dysregulation of SUMO pathways has been implicated in cancer and neurodegenerative disorders, where altered nuclear protein dynamics contribute to disease (Schnur et al., 2021; Yang et al., 2025; Saad et al., 2025; Alkuraya et al., 2006).

Glycosylation is the enzymatic addition of carbohydrate chains (glycans) to proteins, most commonly in the endoplasmic reticulum and Golgi apparatus. N-linked glycosylation occurs on asparagine residues, whereas O-linked glycosylation occurs on serine or threonine residues. Defects in glycosylation pathways cause congenital disorders of glycosylation (CDGs), which are multisystem Mendelian disorders characterised by developmental delay, hypotonia, and organ dysfunction (Vissing et al., 2026; Shwetabh et al., 2025; Polet et al., 2025; Lebon et al., 2025; Kucukcongar Yavas et al., 2025).

Lipidation refers to the covalent attachment of lipid moieties, such as palmitate, myristate, or prenyl groups, to proteins. This modification enhances membrane association and regulates subcellular targeting, signalling complex assembly, and protein stability. Abnormal lipidation contributes to oncogenesis and certain developmental disorders by disrupting membrane signalling pathways (Jiang and Beaudet, 2004; Hornemann, 2015).

ADP-ribosylation is a post-translational modification in which ADP-ribose is transferred from nicotinamide adenine dinucleotide (NAD^+^) to specific amino acid residues on target proteins. PARP-family enzymes mediate many of these reactions, particularly in DNA damage responses. Disruption of ADP-ribosylation pathways is associated with several human diseases, especially neurological disorders involving defective DNA repair. For example, pathogenic variants in *PNKP, APTX, TDP1, and XRCC1* impair single-strand break repair and cause inherited neurodegenerative conditions characterised by cerebellar atrophy and ataxia (Mani et al., 2019; Yoon and Caldecott, 2018).

Histone lactylation is the addition of lactyl groups, derived from intracellular lactate, to lysine residues on histones. This modification links cellular metabolic state to chromatin structure and transcriptional output. Lactate, traditionally considered a by-product of glycolysis, is now recognised as a signalling metabolite capable of influencing gene expression through chromatin remodelling (Lu et al., 2024).

Many post-translational modifications, particularly glycosylation and lipidation, are closely coupled to protein trafficking through the endoplasmic reticulum and Golgi apparatus. Disruption of these modifications not only alters protein stability and activity but also impairs correct intracellular localisation.

### 7.3 Proteolytic processing

Proteolytic processing is an irreversible post-translational modification in which specific peptide bonds are cleaved by proteases to generate mature, active, or functionally distinct proteins. Unlike reversible modifications such as phosphorylation, proteolysis permanently alters protein structure and function. Thus, proteolytic post-translational modification is a tightly regulated mechanism that enables rapid, irreversible control of protein activity, localisation, and signalling (Rogers and Overall, 2013). Proteolytic processing is critical in developmental signalling pathways. In the Notch signalling pathway, ligand binding triggers sequential proteolytic cleavages of the Notch receptor, culminating in release of the intracellular domain, which translocates to the nucleus to regulate gene expression. Disruption of this process, including variants in *NOTCH1*, is associated with congenital heart defects and developmental disorders (Stanley et al., 2024).

Defective proteolytic maturation can also impair extracellular-matrix assembly. In *FBN1*-related Marfan syndrome, abnormal fibrillin processing and assembly disrupt microfibril formation and connective-tissue integrity, contributing to skeletal, ocular and cardiovascular manifestations (Milewicz et al., 2021).

### 7.4 Subcellular localisation and trafficking

Protein function depends on correct subcellular localisation. Newly synthesised proteins must be directed to the appropriate compartment, including the cytosol, nucleus, endoplasmic reticulum, Golgi apparatus, plasma membrane, mitochondria or lysosome. This spatial organisation ensures that proteins encounter the correct binding partners, substrates and regulatory signals.

Proteins destined for secretion or membrane localisation are translated on ribosomes associated with the rough endoplasmic reticulum. Defects in co-translational translocation or ER-associated degradation can lead to accumulation of misfolded proteins within the ER lumen, triggering ER stress and activation of the unfolded protein response. If persistent, this stress response contributes to cellular dysfunction and neurodegeneration.

Mitochondria represent a specialised compartment for protein synthesis, possessing their own translational machinery. Pathogenic variants affecting mitochondrial tRNAs or mitoribosomal proteins impair intramitochondrial translation, resulting in defective oxidative phosphorylation, energy deficiency and predominantly neuromuscular phenotypes (Yarham et al., 2014; Greber and Ban, 2016).

In highly polarised cells such as neurons, spatial regulation is particularly critical, with localised translation and trafficking supporting synaptic function. Disruption of subcellular targeting, intracellular transport or compartment-specific protein synthesis can therefore contribute to neurodevelopmental and neurodegenerative disease.

### 7.5 Protein degradation pathways

Protein degradation pathways remove misfolded, damaged, mislocalised or excess proteins. These systems regulate protein quality, signalling, cell-cycle control and stress responses, while preventing accumulation of toxic aggregates. Two major intracellular systems coordinate this process: the ubiquitin-proteasome system, which primarily degrades short-lived, regulatory and misfolded proteins, and the autophagy-lysosome pathway, which clears long-lived proteins, protein aggregates and damaged organelles. These pathways are especially important in long-lived post-mitotic cells such as neurons, where failure of protein clearance can lead to progressive cellular dysfunction. Disruption of protein degradation can cause disease through abnormal protein persistence, loss of regulated signalling, impaired organelle quality control or toxic aggregate accumulation. These mechanisms are central to neurodevelopmental, neurodegenerative, inflammatory and multisystem disorders.

#### 7.5.1 Ubiquitin-proteasome system (UPS)

The ubiquitin–proteasome system selectively degrades short-lived, regulatory and misfolded proteins. Substrates are tagged through an ATP-dependent cascade in which an E1 enzyme activates ubiquitin, an E2 enzyme carries it and an E3 ligase transfers it to the target protein. Substrate specificity is determined predominantly by the hundreds of E3 ligases, which recognise degradation signals within individual proteins (Jiang and Beaudet, 2004; Komander and Rape, 2012).

The topology of the ubiquitin modification influences substrate fate. K48-linked polyubiquitin chains generally direct proteins to the proteasome, whereas K63-linked chains more often regulate signalling, DNA repair and selective autophagy. Monoubiquitination contributes to trafficking, endocytosis and histone regulation, while mixed and branched chains provide additional regulatory complexity. Deubiquitinating enzymes edit or remove these signals, rescue selected substrates, recycle ubiquitin and regulate commitment to degradation (Komander and Rape, 2012).

Terminal degradation occurs within the 26S proteasome. Its 19S regulatory particle recognises polyubiquitinated substrates, removes ubiquitin, unfolds the protein and translocates it into the 20S catalytic core. The β1, β2 and β5 subunits provide caspase-like, trypsin-like and chymotrypsin-like activities, respectively. During interferon signalling, inducible catalytic subunits form the immunoproteasome, linking protein degradation to antigen processing and immune surveillance (Bard et al., 2018).

The ubiquitin–proteasome system is integrated with broader protein-quality-control pathways. Endoplasmic-reticulum-associated degradation retrotranslocates misfolded secretory proteins for cytosolic disposal; ribosome-associated quality control removes aberrant nascent chains from stalled ribosomes; and the co-chaperone CHIP, encoded by *STUB1*, converts prolonged chaperone engagement of an irreversibly misfolded client into a ubiquitination signal (Trouvé and Férec, 2025; Jiang and Beaudet, 2004).

Failure of this system causes disease through substrate accumulation, defective signalling or impaired clearance of damaged proteins. Loss of neuronal *UBE3A* expression causes Angelman syndrome, although the critical disease-relevant substrates remain incompletely defined. Pathogenic *VHL* variants impair ubiquitination of hypoxia-inducible factors, causing their stabilisation and constitutive hypoxia signalling. In *PRKN*-related Parkinson disease, defective ubiquitination of damaged mitochondria compromises mitophagy and promotes neuronal degeneration. Variants in *FBXO11* disrupt substrate recognition by the SCF ubiquitin-ligase complex, altering developmental signalling and protein turnover (Gregor et al., 2018; Gregor et al., 2025).

Defects in proteasome components and associated quality-control pathways provide further examples. Pathogenic *PSMB8* variants impair immunoproteasome function and cause proteasome-associated autoinflammatory syndromes characterised by accumulation of ubiquitinated proteins and chronic immune activation (Agarwal et al., 2010). In NGLY1 deficiency, failure of cytosolic deglycosylation disrupts processing of retrotranslocated glycoproteins and perturbs endoplasmic-reticulum-associated degradation and proteostasis (Fujihira and Suzuki, 2026).

#### 7.5.2 Autophagy–lysosome pathway

The autophagy–lysosome pathway delivers cytoplasmic material to lysosomes through macroautophagy, microautophagy and chaperone-mediated autophagy. Macroautophagy encloses cargo within a double-membraned autophagosome, microautophagy internalises material through lysosomal membrane invagination, and chaperone-mediated autophagy translocates selected soluble proteins directly across the lysosomal membrane (Levine and Kroemer, 2008).

Macroautophagy proceeds through coordinated membrane initiation, nucleation and elongation. The ULK1 complex initiates phagophore formation, while the class III phosphatidylinositol 3-kinase complex, containing VPS34 and BECN1, generates phosphatidylinositol 3-phosphate and recruits downstream effectors such as WIPI proteins. Expansion and closure of the autophagosomal membrane require the ATG12–ATG5–ATG16L1 complex and LC3 lipidation. The completed autophagosome subsequently fuses with the lysosome, forming an autolysosome in which cargo is degraded by acid hydrolases (Levine and Kroemer, 2008).

Autophagy can be selective as well as bulk-responsive. Cargo receptors including p62/SQSTM1, NBR1, optineurin and NDP52 bind both ubiquitinated substrates and LC3, linking marked cargo to the autophagosomal membrane. This mechanism supports the clearance of damaged mitochondria, protein aggregates, endoplasmic reticulum and intracellular pathogens. Mitophagy is exemplified by the PINK1–parkin pathway, whereas aggrephagy removes protein assemblies that are poorly handled by the proteasome (Levine and Kroemer, 2008; Ding et al., 2007; Kim et al., 2008).

Chaperone-mediated autophagy provides a vesicle-independent route for individual soluble proteins. Substrates containing KFERQ-like motifs are recognised by HSC70, delivered to the lysosomal receptor LAMP2A, unfolded and translocated into the lysosomal lumen (Kaushik and Cuervo, 2018). LAMP2A is one of three isoforms generated by alternative use of the final exon of *LAMP2*, which produces proteins sharing a luminal domain but differing in their transmembrane segment and short cytosolic tail. LAMP2A is the chaperone-mediated autophagy receptor and predominates in liver and fibroblasts; LAMP2B is the isoform predominantly expressed in cardiomyocytes and skeletal muscle, where it supports autophagosome-lysosome fusion through interaction with ATG14 and VAMP8; and LAMP2C mediates lysosomal uptake of nucleic acids. Isoform distribution therefore predicts which tissue is affected when the shared exons are lost (Chi et al., 2019; Qiao et al., 2023). Lysosomal capacity is dynamically regulated by TFEB, which coordinates expression of lysosomal and autophagy genes in response to nutrient availability and cellular stress (Napolitano and Ballabio, 2016).

Disruption of this pathway causes accumulation of undegraded material and progressive cellular dysfunction. Biallelic *EPG5* variants impair autophagosome–lysosome fusion and cause Vici syndrome, a multisystem disorder characterised by neurodevelopmental impairment, cardiomyopathy, immunodeficiency and hypopigmentation (Byrne et al., 2016; Mahjoubi et al., 2022; Piano Mortari et al., 2018). Pathogenic *WDR45* variants impair autophagosome formation and cause β-propeller protein-associated neurodegeneration, with developmental delay followed by progressive neurological decline and basal-ganglia iron accumulation (Xiong et al., 2021).

These disorders illustrate that defects at different stages of autophagic flux produce overlapping but mechanistically distinct phenotypes, emphasising the importance of intracellular trafficking and lysosomal degradation in human disease.

#### 7.5.3 Integration and shared regulation

The ubiquitin–proteasome and autophagy–lysosome pathways are functionally coupled. Under nutrient-replete conditions, mTORC1 suppresses autophagy by phosphorylating ULK1 and retaining TFEB in the cytoplasm. Nutrient withdrawal or energy stress reduces mTORC1 activity, while AMPK activates ULK1, permitting TFEB nuclear translocation and increased autophagic and lysosomal gene expression (Roczniak-Ferguson et al., 2012; Kim et al., 2011). Ubiquitin provides a shared cargo signal between the two pathways. The receptor p62/SQSTM1 binds ubiquitinated substrates and LC3, directing aggregated or proteasome-resistant cargo towards selective autophagy. Proteasome impairment can consequently induce compensatory autophagy through proteotoxic and endoplasmic-reticulum stress, as well as TFEB activation, although this compensation is incomplete and context dependent (Ding et al., 2007; Kim et al., 2008; Li et al., 2019).

The consequences of dysfunction in one pathway therefore depend partly on the residual capacity of the other. This interdependence is particularly important in long-lived post-mitotic cells, which cannot dilute damaged proteins through cell division and are vulnerable to cumulative proteotoxic stress (Ishikawa and Ishikawa, 2020).

#### 7.5.4 Disease mechanisms

Disruption of E3 ligases and deubiquitinating enzymes causes neurodevelopmental disease by altering the abundance or activity of selected regulatory substrates rather than producing global failure of protein degradation. Loss of neuronal expression of the maternal *UBE3A* allele causes Angelman syndrome. Haploinsufficiency of *TRIP12*, loss of *CUL4B* function and pathogenic *USP7* variants cause distinct intellectual disability syndromes, while *USP9X* variants produce sex- and variant-dependent neurodevelopmental phenotypes. The mechanism of *HUWE1*-related disease is more heterogeneous, with both increased dosage and functionally altered missense variants reported, and should not be described uniformly as loss of ligase activity.

Proteasome dysfunction produces both inflammatory and neurodevelopmental disorders. Germline variants affecting immunoproteasome subunits or assembly factors, including *PSMB8*, cause proteasome-associated autoinflammatory syndromes such as CANDLE/PRAAS. By contrast, VEXAS syndrome is not an inherited disorder. It results from acquired somatic *UBA1* variants, most commonly affecting the p.Met41 alternative translation initiation codon, arising in haematopoietic stem cells and restricted to the myeloid lineage. Loss of the cytoplasmic UBA1b isoform impairs ubiquitin activation and activates cellular stress and innate immune pathways. VEXAS is therefore a disorder of somatic mosaicism and of upstream ubiquitin activation rather than a germline proteasome-subunit defect and it is included here to mark that boundary explicitly (Beck et al., 2020). Variants in *PSMD12* and *PSMC3* cause neurodevelopmental proteasomopathies, illustrating how disruption of different proteasome components, inheritance patterns and cellular contexts produces divergent phenotypes.

Defects in the autophagy–lysosome pathway commonly cause multisystem disorders with prominent neurological involvement. Biallelic *EPG5* variants cause Vici syndrome; *WDR45* variants cause β-propeller protein-associated neurodegeneration; and biallelic *ATG7* variants impair canonical LC3 conjugation and autophagic flux. Pathogenic variants in *SPG11*, *ZFYVE26* and *TECPR2* cause hereditary spastic paraplegias associated with autophagic–lysosomal dysfunction. *LAMP2* deficiency causes Danon disease. Because pathogenic variants typically affect shared exons and remove all three isoforms, the phenotype reflects broad impairment of lysosomal and autophagic function; the cardiac and skeletal muscle involvement is attributable principally to loss of LAMP2B-dependent autophagosome maturation and fusion, rather than to failure of chaperone-mediated autophagy alone (Chi et al., 2019; Qiao et al., 2023).

Impaired aggregate and organelle clearance also contributes to adult neurodegenerative disease. Accumulation of tau, α-synuclein, polyglutamine-expanded huntingtin and TDP-43 reflects the combined effects of aggregation, impaired degradation and progressive proteostatic overload. The *C9ORF72* repeat expansion combines toxic RNA and dipeptide-repeat gain-of-function mechanisms with reduced C9ORF72-dependent vesicular trafficking and autophagy; dipeptide-repeat proteins additionally perturb proteasomal and RNA-processing pathways. These disorders predominantly affect neurons because long-lived post-mitotic cells cannot dilute accumulating damage through cell division and are therefore particularly dependent on sustained protein and organelle clearance.

## 8 Integrative synthesis: Regulatory models and regulatory convergence

The regulatory continuum summarised here is not proposed as a new biological model. Multilayer control of gene expression has been established in prior syntheses of transcriptional, chromatin and noncoding-RNA regulation (Lelli et al., 2012; Sibilio et al., 2025; Mattick et al., 2023). Its value in clinical genomics is integrative: it links the position of a molecular lesion to the expected readout, the appropriate tissue and the most informative orthogonal assay.

### 8.1 Four regulatory models linking pathogenic variants to phenotype

The layers described above are more useful clinically when reduced to a small number of reusable templates. Four models account for most of the disorders considered here. They are defined by where the lesion sits and what it does to gene output rather than by which molecular layer it occupies, because several disorders traverse more than one layer. Each model specifies a direction of travel, a defining property and a diagnostic consequence (Figure 5).

**FIGURE 5:**
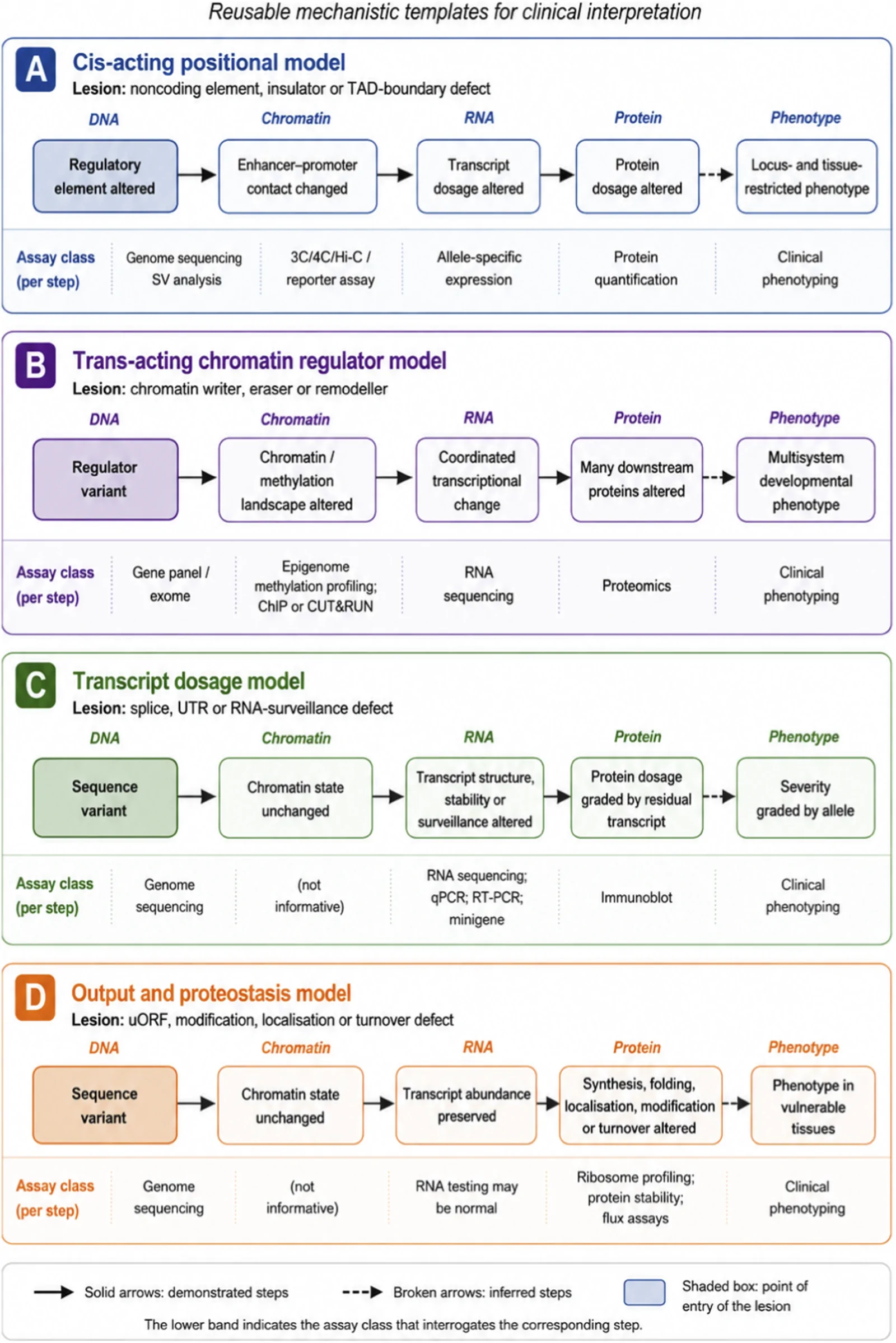
Four regulatory models linking pathogenic variants to Mendelian phenotypes. Each panel shows one model on a common horizontal axis running from DNA through chromatin, RNA and protein to phenotype, so that the four models can be compared directly. The lesion is marked at its point of entry into the pathway. Solid arrows indicate steps for which the mechanism has been demonstrated in patient-derived material or in a model system, and broken arrows indicate steps that remain inferred. The lower band of each panel names the assay class that interrogates the corresponding step. **(A)** Cis-acting positional model, in which a noncoding element or boundary lesion alters enhancer-promoter contact at a structurally intact gene. **(B)** Trans-acting chromatin regulator model, in which a lesion in a broadly expressed chromatin regulator produces coordinated change across many downstream loci and a transferable episignature. **(C)** Transcript dosage model, in which transcript structure, stability or surveillance is altered and severity is graded by residual functional transcript. **(D)** Output and proteostasis model, in which transcript abundance is preserved and the lesion acts on protein synthesis, modification, localisation or turnover, so that RNA-based assays are uninformative.

The first is the cis-acting positional model. A lesion in a noncoding regulatory element, insulator or topologically associating domain boundary alters enhancer-promoter contact and changes transcription of a structurally intact gene. The direction of travel runs from DNA to three-dimensional architecture to transcription to protein dosage to phenotype. Representative examples include the *ZRS* enhancer and *SHH*, boundary disruption at the *EPHA4* locus and deletion of the α-globin regulatory element in α-thalassaemia. The defining property is that the disease gene is not itself mutated, so coding-sequence interpretation is uninformative and the diagnostic readout is allele-specific expression, chromatin conformation or a reporter assay.

The second is the trans-acting chromatin regulator model. A lesion in a broadly expressed chromatin writer, eraser or remodeller alters the histone or DNA methylation landscape and produces coordinated transcriptional change across many target loci. The direction of travel runs from DNA to chromatin state to the transcriptome to phenotype. Representative examples include *KMT2D* in Kabuki syndrome, *EED* in Cohen-Gibson syndrome and *ARID2*. The defining property is that the effect on the mutated gene is direct but the phenotype is mediated indirectly through downstream targets, which is why a peripheral blood episignature can be diagnostic for a phenotype that is developmental and largely neurological.

The third is the transcript dosage model. A lesion alters transcript structure, stability or surveillance and thereby reduces or distorts transcript supply and protein dosage. The direction of travel runs from DNA to RNA to protein dosage to phenotype, with severity graded by residual functional transcript. Representative examples include the β-thalassaemia allelic series, *RNU4-2*, splice variants that trigger nonsense-mediated decay and the *EIF4A3* 5′ UTR expansion described above. The defining property is quantitative dose responsiveness, so a single gene generates graded phenotypes and the informative assay is quantitative rather than qualitative.

The fourth is the output and proteostasis model. Transcript abundance is normal or near normal, but protein output, folding, modification, localisation or turnover is altered. The direction of travel runs from DNA to protein to phenotype, bypassing measurable change in RNA. Representative examples include *NIPBL* 5′ UTR upstream open reading frame variants, hypusination defects in *DHPS* and *DOHH*, *STUB1*, and the ubiquitin-proteasome and autophagy disorders. The defining property is that RNA-based assays are normal, so a negative RNA study does not exclude disease and protein-level evaluation is required.

The models are not mutually exclusive, and combination is informative rather than untidy. The *EIF4A3* expansion is a transcript dosage lesion acting on a factor that itself operates in trans, so it combines the second and third models. *MECP2*-related disease is a chromatin regulator lesion in which the reader rather than the writer of the mark is affected. The models are therefore offered as interpretive templates, and the practical test of a candidate mechanism is whether it can be assigned to one of them, and whether the readout it predicts is observable in a tissue that can be sampled.

### 8.2 Lesion locus and route to phenotype

Two orthogonal distinctions organise the disorders considered here. The first concerns the position of the lesion: cis-acting lesions alter a regulatory element in physical continuity with the affected gene, whereas trans-acting lesions alter a diffusible factor that acts across many loci. The second concerns the route to phenotype: the effect is direct when the phenotype follows from altered function of the mutated gene product itself, and indirect when it follows from altered expression or handling of downstream targets. These distinctions are annotated for each layer in Table 1 and for representative disorders in Supplementary Table S1.

Crossing the two distinctions yields a practical prediction. Trans-acting lesions in broadly expressed regulators act indirectly, produce multisystem phenotypes and generate coordinated, tissue-transferable signatures, which is why episignature testing in peripheral blood is informative in the chromatinopathies despite the phenotype being developmental and largely neurological. Cis-acting lesions act directly on a single locus, produce phenotypes restricted to the tissues in which that element is active and generate no transferable signature, so they require locus-specific and often tissue-specific assays. The distinction therefore predicts assay class before any assay is performed, and a mismatch between the two, for example, a negative episignature in a clinically convincing chromatinopathy, should prompt reconsideration of the lesion class rather than only of the candidate gene.

### 8.3 Regulatory convergence

Regulatory convergence occurs when lesions at different levels produce the same proximate cellular consequence. Reduced protein dosage may result from promoter silencing, aberrant splicing with nonsense-mediated decay, impaired translation initiation or accelerated degradation. Conversely, similar variant classes can produce different phenotypes when they act in different isoforms, lineages or developmental windows. The clinically relevant unit is therefore the variant-mechanism-context combination, not the gene or regulatory layer alone.

The framework is organised around diagnostic decisions: what molecular consequence is predicted; where and when should it be observable; which assay can distinguish the proposed mechanism from alternatives; and what constitutes orthogonal confirmation. Table 4 makes this distinction explicit.

**TABLE 4:** Integrative summary of mechanism-directed interpretation across regulatory layers.

Regulatory layer	Representative mechanisms	Expected molecular consequences	Informative assays/evidence	Key interpretation cautions
Chromatin and 3D genome regulation	Chromatin accessibility; histone or DNA modification; remodelling defects; enhancer, insulator, boundary or loop disruption	Altered dosage, ectopic or reduced expression, abnormal developmental timing, or cell-type-specific regulatory-contact disruption	DNA methylation profiling/episignatures; ATAC-seq; ChIP-based assays; reporter assays/MPRA; 3C/4C/Hi-C or targeted chromatin-conformation methods	Effects may be cell-type and developmental-stage specific. TAD overlap alone is insufficient; assess whether a disease-relevant regulatory contact is plausibly altered
Transcriptional regulation	Promoter defects; transcription-factor dysfunction; enhancer–promoter communication; signal-responsive transcription; defective repression	Altered initiation, occupancy, pathway activation, transcriptional programmes or transcriptional stability	RNA-expression studies; reporter assays; DNA-binding and chromatin-occupancy assays; pathway readouts; single-cell expression where appropriate	Interpret in the context of gene networks, dosage sensitivity, signalling state and cellular composition. Mean expression may not capture altered variability
RNA processing and stability	Splice-site, branch-point or regulatory-element variants; NMD; RNA editing; polyadenylation; RNA-binding proteins; noncoding RNAs; localisation defects	Aberrant isoforms, premature termination, transcript depletion, transcriptome-wide biased mis-splicing or altered localisation	RNA sequencing; RT-PCR; minigene assays; long-read RNA sequencing; allele-specific expression; isoform-specific analysis; localisation studies	Blood may be uninformative because of low expression, alternative isoform use or tissue-specific splicing. NMD inhibition may be required to reveal unstable transcripts
Translational regulation	5′-UTR/uORF variants; altered initiation; codon or elongation defects; stop-codon context; RAN translation; ribosome stalling or RQC dysfunction	Altered protein output, initiation-site choice, translational fidelity, selective transcript vulnerability or toxic translation products	Reporter assays; ribosome profiling; polysome profiling; puromycin-based synthesis assays; targeted proteomics; stress-response studies	Distinguish global translational suppression from transcript-selective effects. Ribosome occupancy does not always equal productive protein synthesis
Post-translational proteostasis	Folding defects; PTM abnormalities; trafficking defects; ubiquitin–proteasome dysfunction; autophagy or lysosomal impairment	Altered activity, localisation, stability, aggregation, degradation or proteostasis stress despite normal RNA abundance	Immunoblot/protein quantification; PTM-specific assays; localisation studies; enzymatic assays; proteasome/autophagy flux assays; proteomics	Protein effects may be highly cell-state dependent. Steady-state abundance alone may miss altered turnover, localisation, complex assembly or stress-induced dysfunction

Cross-cutting principles: distinct molecular lesions may converge on altered dosage, timing, localisation or proteostasis; assay choice should match the suspected mechanism; tissue, developmental stage and isoform context are critical; and a negative blood-based result does not exclude a disease-relevant effect elsewhere.

## 9 Context-specific variant interpretation, regulatory variant classes and functional evidence

Regulatory pathogenicity is conditional. A variant may have a measurable effect only in a particular lineage, developmental stage, activation state or transcript isoform. Neurons are especially vulnerable because they are long-lived, highly polarised and dependent on local translation, axonal transport, synaptic protein turnover and sustained mitochondrial function. They cannot dilute damaged proteins through repeated cell division, and modest defects in proteostasis may accumulate over decades. This helps explain why variants in *FMR1*, *EEF1A2*, autophagy genes and ubiquitin-pathway genes can produce disproportionately neurological phenotypes despite broad expression.

Tissue choice should follow the predicted mechanism. Blood is useful when the gene is expressed and the relevant isoform, pathway and cellular state are represented; it can identify many splice defects, allele-specific expression changes and episignatures. Fibroblasts or myoblasts may be preferable for connective-tissue, mitochondrial or muscle phenotypes. Stimulated lymphocytes may be required for immune-response defects. For brain-restricted genes, induced pluripotent stem cell-derived neurons or direct neuronal models may be justified, although differentiation variability and incomplete maturation limit routine clinical use. Protein or metabolite assays may be more informative than RNA when the defect concerns folding, localisation, enzymatic activity or degradation.

A negative result in blood must therefore be interpreted as ‘not detected in this surrogate under these conditions’, not as exclusion of regulatory pathogenicity. Low expression, alternative isoform usage, nonsense-mediated decay, cell-type dilution and developmental timing can all produce false reassurance. Whenever possible, RNA findings should be paired with an orthogonal readout such as RT-PCR, allele-specific expression, reporter assays, protein quantification, localisation studies, enzymatic testing or rescue experiments. Table 5 summarises a practical tissue-and-assay framework.

**TABLE 5:** Mechanism-directed functional evaluation of regulatory variants.

Variant or mechanism class	Predicted consequence	Detection approach	Functional confirmation	Preferred tissue/model	Major limitation
Promoter or core-promoter variant	Reduced, increased or ectopic transcription initiation	Genome sequencing with promoter annotation; conservation and motif analysis	Reporter assay; allele-specific expression; promoter occupancy or chromatin-accessibility assay	Disease-relevant primary cells; fibroblasts; iPSC-derived cells; stimulated cells where pathway dependent	Reporter systems may not reproduce native chromatin, copy number or long-range regulatory context
Enhancer variant	Altered transcription-factor binding or enhancer activity	Genome sequencing; enhancer maps; activity-by-contact or cell-type-specific regulatory annotations	Reporter assay or MPRA; CRISPR perturbation; target-gene expression; chromatin-conformation assay	Cell type and developmental stage in which the enhancer is active	Enhancer–gene assignment is uncertain; negative testing in an inactive tissue is non-informative
TAD-boundary, insulator or structural variant	Enhancer hijacking, enhancer disconnection, ectopic repression or dosage change	Short- or long-read genome sequencing; optical mapping; SV calling; breakpoint resolution	3C/4C/Hi-C; targeted chromatin-contact studies; expression analysis of candidate target genes	Disease-relevant cells, organoids or engineered models	A variant crossing a TAD boundary is not automatically pathogenic; direction, orientation and cell context matter
Canonical or noncanonical splice variant	Exon skipping, intron retention, cryptic splice-site activation, frameshift or in-frame isoform change	Genome/exome sequencing with splice prediction; branch-point and regulatory-element annotation	RNA sequencing; RT-PCR; long-read RNA; minigene assay; NMD inhibition when appropriate	Tissue expressing the relevant transcript and isoform; fibroblasts, muscle, blood or iPSC-derived cells as appropriate	Minigenes may not reproduce tissue-specific factors or full genomic context; blood can be falsely negative
Polyadenylation or 3′-end-processing variant	Read-through transcription, alternative polyadenylation, altered 3′ UTR and reduced transcript stability	Genome sequencing with 3′-UTR and poly(A)-signal annotation	3′ RACE; long-read RNA sequencing; transcript-length and abundance analysis; reporter assay	Tissue expressing the relevant isoform	Alternative polyadenylation is strongly tissue and developmental-stage dependent
NMD-sensitive nonsense or frameshift variant	Mutant-transcript degradation and haploinsufficiency	Coding-variant detection plus transcript-structure assessment	Allele-specific RNA quantification with and without NMD inhibition; protein quantification	Accessible expressing tissue or patient-derived cells	Absence of mutant RNA may reflect efficient NMD rather than lack of expression; escape rules are transcript dependent
Stop-loss or nonstop-decay variant	C-terminal extension, ribosome stalling, transcript decay or unstable extended protein	Genome sequencing with stop-loss annotation	RNA abundance; ribosome occupancy; immunoblot or targeted proteomics for extended product	Patient-derived cells expressing the gene; engineered cell model if unavailable	A stable transcript does not prove production of a stable extended protein; both RNA and protein should be assessed
miRNA-site or noncoding-RNA variant	Altered post-transcriptional repression or disruption of local regulatory RNA function	Genome sequencing; seed-site or ncRNA structural annotation; copy-number analysis	Dual-luciferase or equivalent reporter assay; AGO-RIP/CLIP where feasible; target RNA and protein quantification	Cell type in which the miRNA/ncRNA and target are co-expressed	RNA-seq alone may miss translational repression; predicted binding changes have high false-positive rates
5′-UTR, Kozak or uORF variant	Altered start-site selection, uORF use, reinitiation or main-ORF translation	Genome sequencing with 5′-UTR, Kozak and uORF annotation	Bicistronic or monocistronic reporter assay; ribosome profiling; polysome profiling; protein quantification	Relevant primary cells or engineered model with the native 5′ UTR	Reporter architecture can create artefacts; transcript abundance may be normal despite large protein effects
Elongation, ribosome-stalling or RQC defect	Slowed elongation, ribosome collisions, aberrant nascent chains or selective translation failure	Gene-based sequencing; repeat-expansion testing where appropriate; proteome and pathway annotation	Ribosome profiling/disome profiling; collision markers; nascent-chain assays; proteostasis and stress-response studies	Neuronal or muscle models; iPSC-derived cells; disease-relevant stress conditions	Basal assays may be normal; defects may emerge only under high translational demand or stress
PTM, trafficking or degradation defect	Altered activity, localisation, stability, aggregation or clearance	Coding-variant detection; domain and pathway analysis; copy-number or repeat-expansion testing as relevant	Immunoblot; enzymatic or PTM-specific assay; microscopy; pulse-chase; proteasome/autophagy flux; targeted proteomics	Disease-relevant cells, organoids or appropriately engineered models	Normal total protein abundance does not exclude abnormal localisation, modification, complex assembly or turnover

A substantial proportion of pathogenic variation lies outside protein-coding sequence. These variants may disrupt gene expression by altering promoter activity, enhancer function, chromatin architecture, splicing, RNA stability, translation initiation or transcript surveillance. Their interpretation requires integration of genomic context, conservation, tissue-specific regulatory data, predicted molecular consequence and functional evidence.

### 9.1 Promoter and enhancer variants

Promoter variants can impair transcription initiation by altering core promoter elements, transcription-factor binding or recruitment of the pre-initiation complex. Enhancer variants can disrupt cell-type- or developmental-stage-specific expression by changing transcription-factor occupancy, chromatin accessibility or enhancer-promoter communication. Structural variants affecting TAD boundaries or insulators may also cause disease through enhancer hijacking or inappropriate gene silencing. These mechanisms are often missed by exome sequencing and are better assessed by genome sequencing, structural-variant analysis, epigenomic annotation and functional reporter or chromatin assays.

### 9.2 Splicing variants

Splicing variants may affect canonical splice sites, branch points, polypyrimidine tracts or auxiliary splicing regulatory elements. They can cause exon skipping, intron retention, pseudoexon inclusion, cryptic splice-site activation, frameshifts or nonsense-mediated decay. Importantly, variants annotated as synonymous, missense or intronic may exert their primary pathogenic effect through altered splicing. RNA sequencing, RT-PCR, minigene assays and splice-prediction tools are therefore essential for evaluating suspected splice-disrupting variants.

### 9.3 Variants affecting RNA stability and miRNA binding

Variants within untranslated regions can alter transcript stability, polyadenylation, RNA-binding protein interaction, microRNA binding or translation efficiency. A 5′ UTR variant may create or disrupt an upstream open reading frame, alter Kozak sequence strength or modify ribosome scanning, while a 3′ UTR variant may change miRNA-mediated repression or transcript decay. Stop-loss variants can activate nonstop decay or generate abnormal C-terminal protein extensions. These mechanisms may produce disease despite preserved coding sequence and may require RNA sequencing, reporter assays, ribosome profiling, polysome profiling or protein-level studies for confirmation.

These principles can be operationalised as a mechanism-directed workflow in which phenotype, variant class, inheritance, tissue relevance and prior molecular findings guide the selection of layer-specific functional investigations (Figure 6).

**FIGURE 6:**
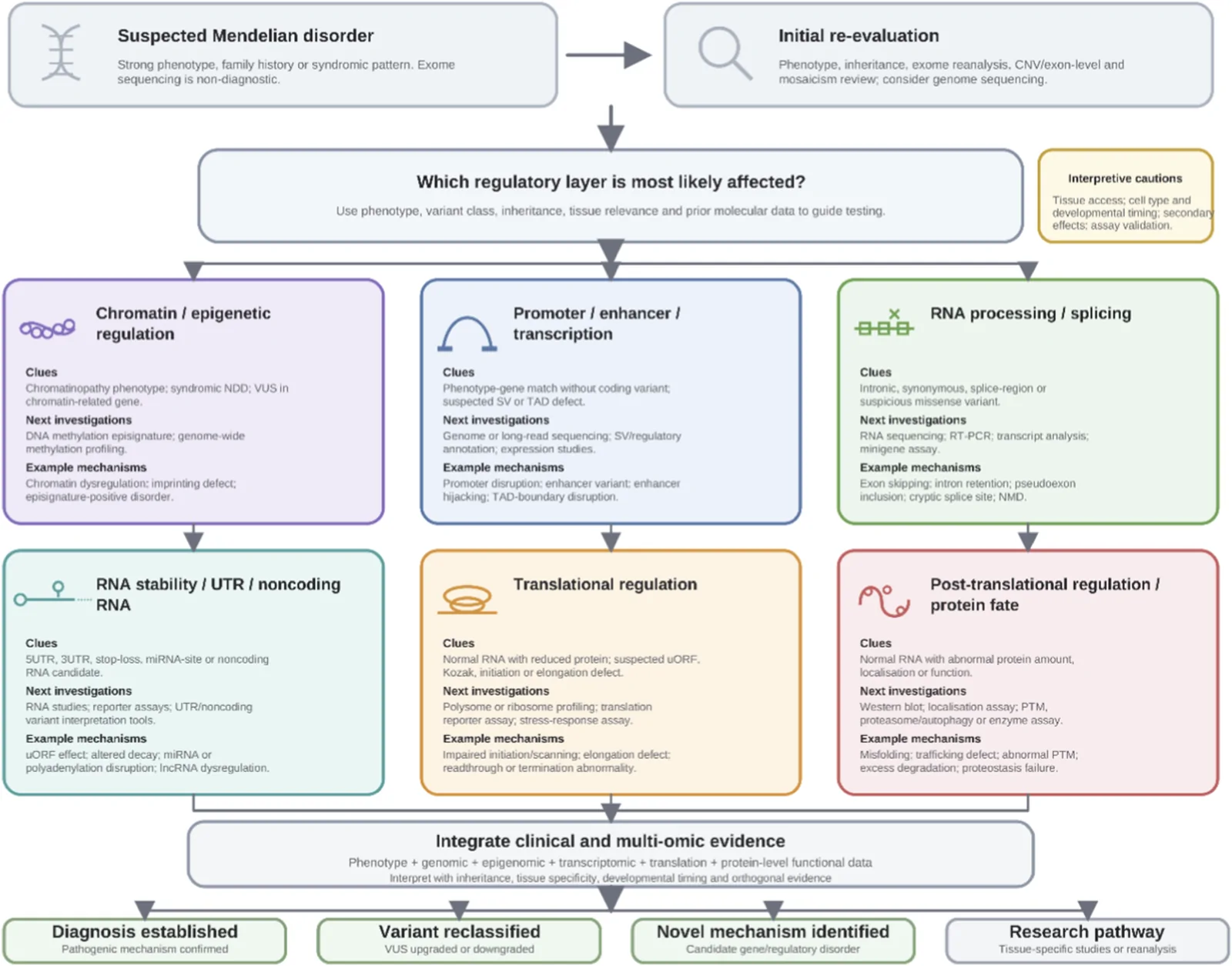
Mechanism-directed diagnostic workflow after non-diagnostic sequencing. Following initial genomic re-evaluation, the suspected regulatory layer guides tissue and assay selection. Genomic and functional evidence is then integrated to support diagnosis, variant reclassification, identification of a novel mechanism or further research. Tissue accessibility, developmental timing, secondary effects and assay validation should be considered when interpreting results.

## 10 Translational implications and concluding perspective

A mechanism-directed classification is therapeutically relevant because regulatory defects may be modifiable even when the underlying DNA variant cannot yet be corrected. Chromatin-directed therapies include inhibitors or activators of histone-modifying enzymes, although broad activity and developmental timing create substantial safety constraints. Splice-switching antisense oligonucleotides can restore exon definition, suppress poison exons or redirect isoform usage; nonsense suppression and NMD modulation are additional strategies, but both require careful assessment of whether the resulting protein is functional or toxic. RNA replacement, small activating RNAs and programmable RNA editing may address transcript deficiency without permanent genome modification.

At the translational level, therapeutic approaches can modify initiation, integrated-stress-response signalling or mTOR activity, while sequence-optimised mRNA can alter transcript stability and ribosome loading. Proteostasis-directed strategies include pharmacological chaperones, proteasome or autophagy modulation, targeted protein degradation and stabilisation of residual protein. The same pathway can require opposite interventions in different disorders: increasing autophagic flux may benefit aggregate accumulation, whereas indiscriminate activation could worsen a primary lysosomal fusion defect.

Clinical translation therefore depends on demonstrating directionality. A functional assay should establish whether disease results from too little, too much, mistimed or mislocalised gene product, and whether correction at the assayed layer restores a disease-relevant phenotype. This requirement connects diagnostic confirmation with therapeutic prioritisation and prevents pathway-level biomarkers from being mistaken for actionable causal mechanisms. Gene regulation extends from chromatin architecture to protein turnover, but the clinically useful insight is not the existence of these layers. It is the ability to formulate a testable chain from variant to mechanism, context, molecular readout and phenotype. Reframed this way, a non-diagnostic sequencing result becomes the starting point for a mechanism-directed workup rather than an endpoint.
