# Supplementary material for: From chromatin to proteostasis: multilevel gene regulation in Mendelian diseases
Source: Front Epigenet Epigenom. 2026 Sep 25;4:1944086. doi: 10.3389/freae.2026.1944086 (PMC13632478; doi:10.3389/freae.2026.1944086)
Supplement: Supplementary file 1 [file Table1.docx]

**Supplementary Table S1. Representative genotype-to-molecular-consequence relationships across regulatory layers**

Representative examples illustrate mechanistic directionality rather than provide an exhaustive catalogue. The table distinguishes experimentally demonstrated molecular links from inferred or proposed links and identifies the principal point at which each causal chain remains uncertain.

**Evidence status:** D = experimentally demonstrated in patient-derived material or an appropriate model system; I = mechanistically inferred or proposed; D/I = a causal chain containing both demonstrated and inferred links; – = no primary effect expected or not applicable. Evidence status refers to the molecular relationships summarised, not to the validity of the gene–disease association. Key reference numbers correspond to the reference list of the revised manuscript.

| **Gene / locus and origin** | **Reference state / pathogenic alteration and genotypic context** | **RNA-level consequence** | **Protein-level consequence** | **Phenotype** | **Mechanistic evidence status** | **Principal inference boundary** | **Key ref(s)** |
| --- | --- | --- | --- | --- | --- | --- | --- |
| ***ZRS / SHH*** *cis* | Reference: intact ZRS–SHH regulatory architecture. Pathogenic: enhancer SNV or structural rearrangement; usually heterozygous, with selected dosage-dependent alleles. | Ectopic or reduced *SHH* transcription in limb-bud context. | Altered SHH signalling output. | Preaxial polydactyly / limb malformation. | **D**: enhancer lesion → altered regulatory activity / SHH expression. **D/I**: exact expression change → phenotype relationship varies by allele and developmental context. | For some structural variants, the altered regulatory contact and quantitative SHH dosage in the relevant embryonic tissue are inferred from architecture rather than measured directly. | 63, 64 |
| **α-globin regulatory region** *cis* | Reference: intact major α-globin regulatory element. Pathogenic: deletion of HS-40 / major α-globin regulatory region; phenotype is allele-dose dependent. | Reduced *HBA1/HBA2* transcription. | Reduced α-globin chain production. | α-thalassaemia spectrum. | **D**: regulatory deletion → reduced α-globin transcription → reduced chain output; human expression evidence supports the causal chain. | Residual phenotype is modified by allelic dosage and background globin genotype, but the core molecular mechanism is directly established. | 65 |
| ***KMT2D*** *trans chromatin regulator* | Reference: normal KMT2D dosage and enhancer-associated COMPASS activity. Pathogenic: heterozygous loss-of-function / haploinsufficiency. | Coordinated dysregulation of enhancer-linked transcriptional programmes. | Distributed alteration of downstream protein dosage and pathway activity. | Kabuki syndrome. | **D**: KMT2D loss → altered enhancer chromatin and transcription. **I**: the full set of downstream protein changes that mediate individual clinical features. | The bridge from broad enhancer/transcriptome dysregulation to specific organ-level phenotypes is distributed and incompletely mapped. | 40-42 |
| ***RNU4-2*** *trans spliceosomal RNA* | Reference: functional U4-2 snRNA within the major spliceosome. Pathogenic: recurrent heterozygous de novo variants in constrained U4-2 regions. | Biased transcriptome-wide mis-splicing. | Distributed downstream isoform and protein-dosage effects. | Severe neurodevelopmental disorder. | **D**: variant → spliceosomal dysfunction → transcriptome-wide mis-splicing. **I**: aggregate downstream proteomic consequences and relative contribution of individual mis-spliced targets. | No single aberrant transcript explains the phenotype; the causal contribution of individual downstream targets remains incompletely resolved. | 121-123 |
| ***HBB* poly(A) signal** *cis* | Reference: normal β-globin 3′-end processing. Pathogenic: polyadenylation-signal substitution; recessive / allele-dose dependent. | Read-through and abnormal 3′-end formation with reduced normal transcript. | Reduced β-globin output. | β-thalassaemia. | **D**: variant → abnormal RNA processing → reduced normal transcript → reduced β-globin output; demonstrated in patient material. | Phenotypic severity remains influenced by residual β-globin production and modifying globin alleles, rather than uncertainty in the primary mechanism. | 87-89 |
| ***EIF4A3*** *cis lesion affecting trans factor* | Reference: adequate EIF4A3 expression and EJC function. Pathogenic: biallelic 5′ UTR repeat expansion; rare missense allele reported in trans. | Reduced *EIF4A3* transcript abundance. | Reduced EJC component dosage / altered EJC-dependent RNA metabolism. | Richieri-Costa-Pereira syndrome. | **D**: repeat expansion → reduced EIF4A3 RNA. **D/I**: reduced dosage → specific downstream EJC defects and developmental phenotype. | Disease-relevant downstream EJC targets and the relative contribution of splicing versus RNA-surveillance defects remain incompletely defined. | 36, 94 |
| **THPO uORF** *cis* | Reference: inhibitory uORF limits THPO translation. Pathogenic: uORF-disrupting variant causing dominant hereditary thrombocythaemia. | Transcript abundance may be largely preserved. | Increased thrombopoietin translation / protein output. | Hereditary thrombocythaemia. | **D**: uORF disruption → translational derepression → increased THPO output; the central molecular mechanism is experimentally established. | The principal uncertainty is quantitative: variant-specific effects on translational efficiency and final platelet phenotype may differ. | 136 |
| ***NIPBL* 5′ UTR** *cis* | Reference: intact 5′ UTR translational control. Pathogenic: heterozygous 5′ UTR variants associated with Cornelia de Lange syndrome. | RNA abundance may be normal or only subtly altered. | Reduced or otherwise altered NIPBL protein output is proposed for translationally disruptive alleles. | Cornelia de Lange syndrome. | **D/I**: human variant–phenotype association is established; the precise RNA → translation → protein consequence is not equally demonstrated for every reported 5′ UTR allele. | The mechanistic effect of individual 5′ UTR variants should not be assumed solely from position; direct translation/protein assays are needed for allele-specific confirmation. | 137, 138 |
| ***C9ORF72*** *cis with downstream trans toxicity* | Reference: non-expanded repeat. Pathogenic: dominant GGGGCC repeat expansion. | Repeat RNA, RNA foci and sense/antisense repeat transcripts. | RAN-translated DPRs with downstream translation, RNA and proteostasis stress. | ALS / frontotemporal dementia. | **D**: expansion → repeat RNA / foci and DPR production. **D/I**: contribution of individual downstream toxic pathways to neuronal degeneration. | RNA toxicity, DPR toxicity, altered C9ORF72 function and proteostasis defects coexist; their relative contributions vary by model, cell type and disease stage. | 154 |
| ***UBE3A*** *trans E3 ligase* | Reference: maternal neuronal UBE3A expression with normal ubiquitin-ligase activity. Pathogenic: loss of maternal neuronal UBE3A function; parent-of-origin dependent. | *UBE3A* RNA may be preserved or reduced depending on variant class and imprinting context. | Loss or alteration of UBE3A E3-ligase function and substrate regulation. | Angelman syndrome. | **D**: parent-of-origin neuronal UBE3A loss and disease association. **D/I**: specific substrate abnormalities responsible for individual phenotypic features. | The critical disease-relevant neuronal substrates and their relative contribution to phenotype remain incompletely defined. | 54, 164 |
| ***EPG5*** *trans autophagy machinery* | Reference: intact EPG5-dependent autophagosome–lysosome fusion. Pathogenic: biallelic loss-of-function variants. | No primary RNA-dosage mechanism is required. | Impaired autophagic flux and cargo clearance. | Vici syndrome. | **D**: EPG5 deficiency → impaired autophagic flux / fusion; cellular and disease-model evidence supports the core mechanism. | The pathway from impaired flux to the full multisystem phenotype is pleiotropic, and tissue-specific downstream injury mechanisms remain incompletely resolved. | 200-202 |
| ***UBA1*** *trans ubiquitin activation* | Reference: normal cytoplasmic UBA1 activity. Pathogenic: acquired somatic myeloid UBA1 variant; mosaic adult-onset disorder. | No primary transcript-dosage mechanism; variant alters UBA1 isoform/function in affected hematopoietic cells. | Reduced cytoplasmic ubiquitin activation with proteotoxic and inflammatory stress. | VEXAS syndrome. | **D**: somatic variant → impaired cytoplasmic ubiquitin activation and cellular stress. **D/I**: full pathway from proteostasis disturbance to the heterogeneous inflammatory phenotype. | The relative contributions of proteotoxic stress, innate immune activation and lineage-specific effects to individual clinical manifestations remain under study. | 208 |

**Abbreviations:** D, demonstrated; I, inferred; EJC, exon-junction complex; DPR, dipeptide repeat protein; ALS, amyotrophic lateral sclerosis; FTD, frontotemporal dementia; uORF, upstream open reading frame.
